# Antimicrobial Resistance and Recent Alternatives to Antibiotics for the Control of Bacterial Pathogens with an Emphasis on Foodborne Pathogens

**DOI:** 10.3390/antibiotics12020274

**Published:** 2023-01-30

**Authors:** Yosra A. Helmy, Khaled Taha-Abdelaziz, Hanan Abd El-Halim Hawwas, Soumya Ghosh, Samar Sami AlKafaas, Mohamed M. M. Moawad, Essa M. Saied, Issmat I. Kassem, Asmaa M. M. Mawad

**Affiliations:** 1 Department of Veterinary Science, College of Agriculture, Food and Environment, University of Kentucky, Lexington, KY 40546, USA; 2 Department of Zoonoses, Faculty of Veterinary Medicine, Suez Canal University, Ismailia 41522, Egypt; 3 Department of Animal and Veterinary Sciences, Clemson University, Clemson, SC 29634, USA; 4 Department of Genetics, Faculty of Natural and Agricultural Sciences, University of the Free State, Bloemfontein 9301, South Africa; 5 Molecular Cell Biology Unit, Division of Biochemistry, Department of Chemistry, Faculty of Science, Tanta University, Tanta 31511, Egypt; 6 Faculty of Medicine, Minya University, Minya 61742, Egypt; 7 Chemistry Department, Faculty of Science, Suez Canal University, Ismailia 41522, Egypt; 8 Institute for Chemistry, Humboldt Universität zu Berlin, Brook-Taylor-Str. 2, 12489 Berlin, Germany; 9 Centre for Food Safety, Department of Food Science and Technology, University of Georgia, Griffin, GA 30609, USA; 10 Department of Biology, College of Science, Taibah University, Madinah 42317, Saudi Arabia; 11 Botany and Microbiology Department, Faculty of Science, Assiut University, Assiut 71516, Egypt

**Keywords:** antimicrobial resistance, antibiotics alternatives, quorum sensing, prebiotics, probiotics, small molecules, antimicrobial peptides, bacteriophage, essential oils, fecal transplant, nanoparticles, organic acids, vaccines, antibodies

## Abstract

Antimicrobial resistance (AMR) is one of the most important global public health problems. The imprudent use of antibiotics in humans and animals has resulted in the emergence of antibiotic-resistant bacteria. The dissemination of these strains and their resistant determinants could endanger antibiotic efficacy. Therefore, there is an urgent need to identify and develop novel strategies to combat antibiotic resistance. This review provides insights into the evolution and the mechanisms of AMR. Additionally, it discusses alternative approaches that might be used to control AMR, including probiotics, prebiotics, antimicrobial peptides, small molecules, organic acids, essential oils, bacteriophage, fecal transplants, and nanoparticles.

## 1. Introduction

Antimicrobial resistance (AMR) is a major public health concern worldwide [1]. Infections with antibiotic-resistant pathogens have a negative influence on the health of humans and other animals because they increase the risk of treatment failures and illness severity [2,3]. Over the past few decades, the misuse of antibiotics in both humans and food-producing animals has resulted in the emergence and dissemination of antibiotic-resistant bacteria [4,5,6]. Between 2000 and 2010, 76% of the global increase in antibiotic use was reported in BRICS countries (Brazil, Russia, India, China, and South Africa) [7]. For example, in 2010, India was the largest antibiotic consumer (12.9 × 10^9^ units; 10.7 units/person), followed by China (10.0 × 10^9^ units; 7.5 units per person), then the US (6.8 × 10^9^ units; 22.0 units per person) [8]. Between 2000 and 2015, global antibiotic use significantly increased by 65% (21.1–34.8 billion) DDDs (defined daily doses), especially in low- and middle-income countries [9]. In the US, approximately 80% of antibiotics are used in livestock production [10]. The uses of sub-therapeutic doses of antibiotics in food-producing animals as growth promoters, therapeutic doses for control, and treatment of infectious diseases [11,12] have also contributed to the development of antimicrobial-resistant microorganisms. Transmission of resistant bacteria from food-producing animals to humans through direct contact, handling, or eating their product poses a substantial threat to human health [13,14]. Antibiotic resistance illnesses currently cause approximately 1.2 million deaths worldwide [15]. However, if no steps are taken to control the spread of antibiotic resistance, the estimated number of deaths will rise to 10 million, with an economic loss of more than $100 trillion by 2050 [15,16]. In the US, more than two million infections with antibiotic-resistant bacteria occur each year; with ~$20 billion in economic losses [16]. Notably, foodborne illnesses caused by *Campylobacter*, *Salmonella*, *E. coli* O157, *Listeria monocytogenes*, *Staphylococcus aureus*, and *Clostridium perfringens* can affect one in six people annually, leading to approximately 128,000 hospitalizations and 3000 deaths, with about $90 billion in the US [17,18]. Many of these pathogens are on the global priority pathogens list of antibiotic-resistant bacteria provided by the National Institute of Health (NIH) and World Health Organization (WHO) [19,20]. Therefore, there is a critical need to control AMR pathogens. In this review, we will highlight the evolution of the AMR problem, the mechanism of acquiring resistance, and the novel non-antibiotic approaches that can be used for reducing the burden of antimicrobial-resistant pathogens.

## 2. Evolution, Source, and Transmission of AMR

**Figure 1:**
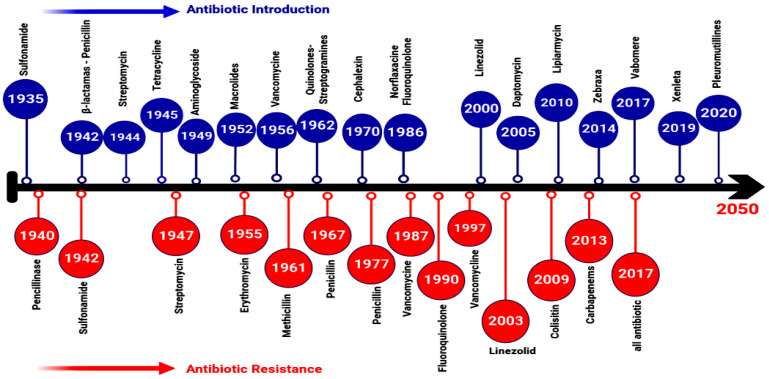
Timeline illustrates antibiotics evolution.

The non-prudent use of antibiotics in livestock production has resulted in an alarming surge of antibiotic-resistant pathogens [21]. The first effective antimicrobial sulfonamide drug Prontosil was discovered in 1932 and approved for human use in 1935 [22]. Resistance to sulfonamide was observed in 1939 due to limitations in safety and efficacy [23]. Despite compelling evidence that the discovery of antimicrobials was revolutionary for controlling many serious and life-threating diseases, one of the major shortcomings associated with their prolonged use is that many pathogenic bacteria may develop or acquire resistance traits over time through a large variety of mechanisms [24]. For example, *S. aureus* was initially sensitive to penicillin; however, it became resistant over time due to the development and production of penicillinase that inactivates the inhibitory impact of penicillin [21]. The evolution and resistance acquisition of different antibiotics is shown in Figure 1. The FDA is carefully seeking to produce new antibiotics to overcome microbial resistance. Eravacycline dihydrochloride (Xerava) is a new synthetic fluorocycline belonging to tetracycline-class antibiotics that was discovered in 2018 [25,26]. It has potent antibacterial activity against Gram-negative and Gram-positive bacteria that usually have tetracycline-specific resistance mechanisms [27]. It inhibits bacterial growth by binding to the bacterial 30S ribosomal subunit [26]. Additionally, levonadifloxacin L-arginine tetrahydrate and the combination of levonadifloxacin and lipoglycopeptide dalbavancin were approved in 2019 by the FDA as antibiotics to treat acute bacterial skin and skin structure infections (ABSSSI) [28]. In 2020, the FDA approved pretomanid in combination with bedaquiline and linezolid for the treatment of drug-resistant *Mycobacterium tuberculosis* [29] and approved remdesivir in combination with baricitinib for the treatment of other pathogens [30,31]. In 2021, ozenoxacin was approved for the treatment of impetigo and bacterial infections caused by *S. pyogenes* or *S. aureus* in children [32,33] and rifapentine for the treatment of tuberculosis [33].

AMR can be caused by (1) microbial genetic mutations [34], (2) incomplete courses of antibiotic, enabling some pathogens to survive and develop resistance to antibiotics [35], (3) overuse of antibiotics [36], (4) using antibiotics at doses lower than recommended, (5) consumption of animal products containing antibiotic residues and (6) using antibiotics containing fertilizers in agriculture and/or animal farming [37]. The continuous environmental overlapping between livestock and human activity provides many opportunities for the transmission of antimicrobial-resistant bacteria or development of their AMR genes in both directions [38]. AMR can be transmitted through (1) direct close contact between human and livestock [39,40], (2) ingestion of contaminated food and water, (3) contaminated bio wastes, (4) transportation/importation of animal products across the world, (5) clonal transfer of resistance bacteria or horizontal transmission of AMR genes [36], (6) soil, manure of animals, waste water and sewage, or vectors such as invertebrates (insects and bugs) and wild animals [41,42].

## 3. Mechanism of Acquiring AMR

**Figure 2:**
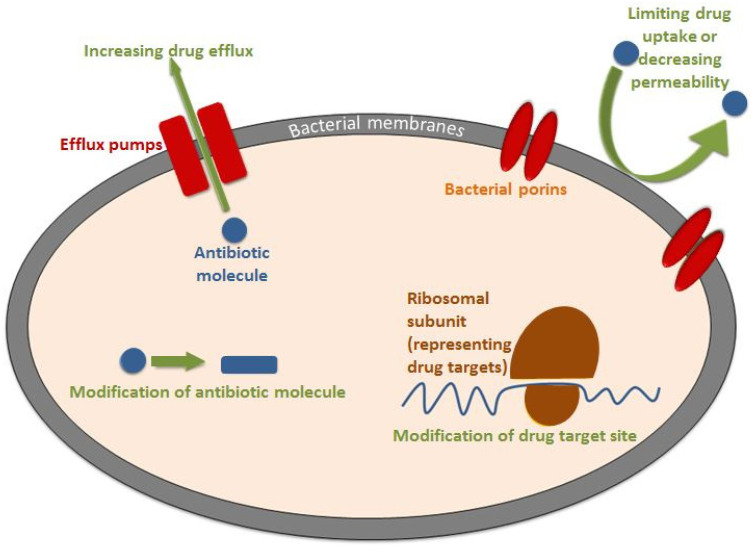
Mechanisms of antimicrobial resistance in bacterial cells.

Antibiotic resistance (AR) in the bacteria can be intrinsic or acquired. Intrinsic resistance is seen in naturally resistant bacteria that exhibit certain inherited properties. For example, the presence of lipopolysaccharide (LPS) in the cell walls of Gram-negative bacteria provides an innate barrier against the penetration of antimicrobials [43]. This intrinsic resistance includes limiting the drug’s uptake and increasing its efflux or inactivation [44]. Meanwhile, acquired antibiotic resistance mechanisms include modification of drug targets, drug efflux, and inactivation [44]. In addition to the aforementioned mechanisms, adaptive mutations are exhibited by bacteria in response to the use of antibiotics as a means of resistance [45,46]. Prior studies reported that adaptive resistance is responsible for in vitro and in vivo differences in antibiotic effectiveness and the failure of clinical antibiotic therapies [24]. Bacterial genetic plasticity aids the acquisition of AR. It appears as either mutations in bacterial genes or gain of foreign DNA fragments coding resistance determinants through horizontal gene transfer (HGT) of antibiotic resistance genes (ARGs) [47]. Antibiotic-sensitive bacteria acquire resistance-modulating genes through HGT, which enables bacteria to share their genetic material by one of three techniques, including transduction, conjugation, and transformation [24]. Regarding transduction, the genetic material from a donor-resistant bacterium is transferred to another bacterium by a bacteriophage, a virus that infects and replicates inside bacterial cells. Basically, the bacteriophage attaches to the donor bacterium and injects its genetic material into it, which incorporates itself within the bacterial genome; by the replication of the bacteriophage, multiple bacteriophages are produced carrying genomes containing resistance genes. When the newly created bacteriophage infects another bacterium, it injects the resistance-genes-containing genome into it [48]. Conjugation, which is referred to as bacterial sex, occurs when the donor bacterial cell, containing a resistance-gene-encoding plasmid, attaches to the recipient bacterium. This process requires a pilus (physical attachment between bacterial cells through which the plasmid is transferred) and a type IV secretion system to be accomplished successfully [49]. Unlike transduction and conjugation, transformation occurs after the death of bacteria carrying resistance genes. By bacterial lysis, genetic material is released and naked DNA is picked up by another bacterium and is incorporated into its genome [50]. After bacteria acquire resistance genes by any of the aforementioned mechanisms, genes are expressed in one or more of the following ways (Figure 2):

### 3.1. Limiting Drug Uptake and Decreasing Permeability

#### 3.1.1. Lipopolysaccharide (LPS) of Outer Bacterial Membrane

Bacterial LPS is a conserved major biologically active component of bacterial outer membranes, primarily in Gram-negative bacteria. LPS has a direct role in AMR as a physical barrier limiting the penetration of antimicrobials into bacterial cells. This may explain, at least in part, why-Gram positive bacteria have a lower ability to limit the antimicrobials’ uptake than Gram-negative ones [51].

#### 3.1.2. Bacterial Porins

Bacterial porins are membrane protein channels present in the outer membrane of Gram-negative bacteria. They modulate crossing of hydrophilic molecules, including hydrophilic antibiotics such as β-lactams [52]. The bacteria can also limit drug uptake via the modification of their porin channels, either by decreasing their numbers, as in *Enterobacteriaceae* resistance to carbapenems, or by alteration of porin’s selectivity through mutation, as in resistance of *Neisseria gonorrhoeae* to β-lactam and tetracycline and resistance of *Enterobacter aerogenes* to imipenem and some cephalosporins [44]. This clearly indicates that the outer membrane of Gram-negative bacteria contains overlapping defensive systems that confer not only protection against antibiotics but also to the host antimicrobial factors.

#### 3.1.3. Biofilm Formation

Biofilm is the aggregation of bacterial cells in the form of clusters. Bacterial cells piled up on one another prevent access of antimicrobials into the bacterial cells [53]. The capacity for biofilm formation is widely distributed in bacteria, with approximately 40–80% of terrestrial cells existing in biofilms as a defense mechanism [54]. Biofilm formation supports antimicrobial resistance either directly, by acting as a physical barrier, or indirectly, by facilitating horizontal transfer of resistance genes amongst bacterial cells by conjugation, transduction, and transformation [54]; this is in addition to protection against the host’s immune defenses. Bacteria are able to develop biofilms on inanimate surfaces as well as inside living tissues [55]. For example, the resistance of cystic fibrosis of the lung (caused by *Pseudomonas aeruginosa* infection) to antibiotics is thought to be due to bacterial biofilm formation. Mechanical disruption or removal of biofilms will therefore improve the action of antibiotics by exposing the causative agent to their effects [56,57].

### 3.2. Enzymatic Destruction of Antibiotic Molecules

Resistant bacteria produce antibiotic-degrading enzymes which destroy certain sites in the antibiotic, rendering it ineffective. These enzymes work by acetylation, phosphorylation, glycosylation, or hydroxylation of certain sites in the antibiotic molecule that interfere with the binding of the corresponding drugs to their ribosomal targets [45]. For example, β-lactams-hydrolyzing enzymes impede the ability of β-lactams to inhibit bacterial cell wall biosynthesis [58]. Additionally, macrolides are encountered by resisting bacteria through modification of macrolide esterases (Eres) and macrolide phosphotransferases (MPHs) enzymes [59]. Aminoglycoside (AGS)-resistant bacteria encode aminoglycoside-modifying enzymes (AMEs) in their chromosomes or in mobile genetic elements (MGEs), which is the most commonly utilized mechanism by bacteria for battling the effect of AGS [60].

### 3.3. Drug Target Site Modification

Resistant bacteria inhibit antimicrobials from binding by altering their antimicrobial’s target proteins. It was noticed that most of the bacterial genes involved in these changes are encoded on mobile genetic elements (MGEs) [45]. The changes can be achieved in several ways, including replacement of target sites, enzymatic alterations of binding sites, and mutations in genes encoding target sites [61]. Vancomycin-resistant *S. aureus* is an example of resistance by replacement of a target site, where it replaces the alanine subunit in its cell wall peptides, the target site of vancomycin, with a lactate subunit to which vancomycin cannot bind properly. Eventually, the cross-linking enzyme of the bacterial cell wall can fit into its site and perform its function [62]. Furthermore, macrolides-resisting bacteria showed enzymatic alterations of their binding sites by methylation of the 50S ribosomes, macrolides targets. They carry erm (erythromycin ribosomal methylation) genes, which encode an enzyme responsible for catalyzing the methylation process. The methylated ribosome becomes an unfit target for macrolide antibiotics, thus preventing antibiotic access into the bacterial cell wall [45]. Also, rifampicin-resistant bacteria mutate the rpob gene, which encodes the β subunit of DNA-dependent RNA polymerase (RNAP) that carries the rifampicin binding site, resulting in the substitution of an amino acid in the RPOB protein. Overall, mutations in genes’ encoding target sites decrease the affinity of antibiotics to their targets inside bacteria [63].

### 3.4. Antibiotic-Specific Efflux Pumps

The plasma membranes of bacteria carry protein structures called bacterial efflux pumps (EPs) [64]. These pumps can recognize foreign structures accessing the bacterial cells through their cell wall and pump them out, thus preventing their intracellular accumulation and interaction with their target cells [65]. Efflux pumps can be either substrate-specific for certain antibiotic families or have broad antibiotic activity as in multi-drug-resistant bacteria [66]. However, single efflux pumps may target multiple antibiotics [67]. Thus, multi-drug resistance (MDR) can be achieved by bacteria through either expression of a single efflux pump or overexpression of multiple efflux pumps as in *P. aeruginosa* [68] and the SmeDEF or SmeVWX efflux systems of *Stenotrophomonas maltophilia* [69]. There are five families of efflux pumps, including small multidrug resistance (SMR), resistance-nodulation-division (RND), multidrug and toxic compound extrusion (MATE), the major facilitator superfamily (MFS), and the ATP-binding cassette (ABC) families [70]. All types are found in bacteria, except for the RND family, which is exclusive to Gram-negative bacteria. The over-expression of efflux pumps is associated with clinical antibiotic resistance [71].

## 4. Novel Strategies to Combat AMR

### 4.1. Small Molecules (SMs)

SMs are non-peptide organic molecules that are synthetic or obtained from natural product extracts. They have drug-like properties that can interact with biological molecules, including protein and nucleic acids, and can alter their normal functions. The low molecular weight (~200–500 Da) and high hydrophilicity of these molecules allow their effective absorption by both host and pathogen barriers [72,73]. SMs can be modified to enhance the qualities desired for specific applications, such as stability and solubility under adverse environmental conditions. These properties can be exploited to enhance the SMs’ antimicrobial efficacy and their mass applicability. High-throughput screening (HTS) of SM libraries is commonly used for the development of antibacterial drugs and identification of SM candidates that inhibit either bacterial growth in whole-cell assays or the activity of a main bacterial enzyme or protein [74]. Indeed, a cost-effective, cell-based HTS expedient approach has been recently developed to enhance anti-bacterial molecule discovery [75,76]. A summary of the SMs identified using HTS is shown in Table 1. SM antimicrobials targeting bacterial membranes are highly desired because they have low potential for resistance development by pathogens, can potentiate the activity of many antibiotics, are effective against slow-growing bacteria and biofilms, and have high stability in serum and good tissue penetration [77]. They have been reported to be effective against several MDR bacteria, such as *E. coli*, *P. aeruginosa*, *Enterococcus faecium*, methicillin resistant *S. aureus* (MRSA), *Klebsiella pneumoniae*, *Acinetobacter baumannii*, and *Enterobacter* species [78,79,80]. Recently, SMs were used for the treatment of plant pathogens such as *Xanthomonas* spp., *Erwinia tracheiphila*, *Acidovorax citrulli* and *Salmonella* infection [81,82,83,84], as well as for the potentiation of antibiotics, which can help in reducing the resistance of the treated bacteria [85].

#### 4.1.1. Mechanisms of Actions of SMs

The low molecular weights of SMs facilitate their infiltration into target cells [86]. Following infiltration, SMs interfere with or inhibit certain molecules involved in cellular pathways [87]. Examples of these cellular pathways include (1) biosynthesis of microbial cell envelopes that can be inhibited by SMs through inhibition of the dephosphorylation of the central lipid carrier undecaprenyl pyrophosphate (C_55_-PP) to C_55_-P, such as THCz, which in turn, interferes with lipid II (peptidoglycan), lipid III_WTA_ (wall teichoic acid), and lipid I_cap_ (capsule), involved in cell envelope biosynthesis pathways, (2) interference with bacterial cell division through inhibition of a key component in that process, FtsZ [88], and (3) encountering quorum sensing through inhibition of LsrK, which is an essential component for initiation of the QS cascade [89].

#### 4.1.2. Limitations of SMs

Despite their beneficial effects, there are functional limitations of SMs. Limitations include their action inside the recipient’s body, irrespective of the physiologic status. The likelihood of binding to non-target molecules inside the human body leads to undesirable effects [90]. In addition to these limitations, there are also some structural design constrains, such as the difficulty of designing SMs to target unstructured disordered polypeptide, as well as their low affinity to bind or modify the relatively flat protein surface mediators [91]. Moreover, it is challenging to accurately determine the modulating proteins to be targeted by SMs [91].

**Table 1:** Identified SMs against different pathogens and their potential targets.

Bacteria	Name of the SMs	Evaluation	Targets	References
*M. tuberculosis*	Benzimidazole and nitro-triazole	In vitro	Inhibit cell wall biosynthesis	[92]
Uropathogenic *E. Coli*	120304 and 175472	In vitro	TonB system	[93]
*E. coli* and *P. aeruginosa*	Nitrofurans	In vitro	Inhibit bacterial growth through reduction of the nitro group to an amine, followed by damage to bacterial DNA	[94]
*P. aeruginosa* and *S.* Typhimurium	Class 2,4-disubstituted-4H-[1,3,4]-thiadiazine-5-ones, Fluorothiazinon (FT)	In vitro, in mice	Suppress T3SS	[95,96]
*Bacillus subtilis*	Adamantane derivatives (T6102)	In vitro	Inhibit bacterial protein synthesis and bacterial growth	[97]
*S. aureus* and *S. epidermidis*	3-methoxybenzamide derivatives (PC190723)	In vitro (CD-1 mouse hepatocytes), in mice	Disrupt FtsZ	[98]
*S. aureus*	ZY-214-4 (C_19_H_11_BrNO_4_)	In vitro	Suppress biofilm formation	[99]
*Mycoplasma gallisepticum*	SM4 and SM9	In vitro, in chickens	Alter cell membrane conformation	[76]
*M. bovis*	Methanesulphonic acid, 3-[(2E)-3-(3,4-dihydroxyphenyl) prop-2-enoyloxy](1S,3R,4R,5R)- 1,4,5-trihydroxycyclohexane carboxylic acid, S-carboxymethyl-l-cysteine, l-aspartic acid, dihydrotachysterol, eriodictyol and (+)-a-tocopherol acid succinate)	In vitro	NI*	[100]
*C. jejuni*	Campynexin A	In vitro, in chickens	Inhibit flagellar expression	[101]
*C. jejuni*	Piperazine, aryl amine, piperidine, sulfonamide and pyridazinone molecules	In vitro	NI*	[75]
*C. jejuni*	TH-4 and TH-8	In vitro, in chickens	Alter cell membrane integrity	[102]
*S.* Typhimurium	JD1	In vitro, in mice	Inhibit bacterial growth by distorting cytoplasmic membranes through increasing fluidity and disrupting barrier function	[103]
*S.* Typhimurium	Imidazole and methoxybenzylamine	In vitro, *Galleria mellonella* larvae, in chickens	Alter cell membrane integrity	[104]
*Avian pathogenic E. coli* (APEC)	QSI-5 and GI-7	In vitro, *Galleria mellonella* larvae, in chickens	Inhibit quorum-sensing autoinducer-2 and outer membrane proteins	[105,106,107,108]
*E.* faecium	6-indolyl compounds	In vitro	NI*	[109]
*Clostridium difficle* R20291	2-aminoimidazole (2-AI)	In vitro	NI*	[110]
Chlamydia	INPs (Innate Pharmaceuticals AB)	Epithelial cells	Supress Type III secretion	[111]
*Clostridium botulinum*		In vitro, In mice	Inhibit neurotoxin serotype A	[112]
*L. monocytogenes*	Pimozide (antipsychotic drug)	In vitro: murine bone marrow-derived macrophages (BMM)	Decrease the vacuole escape and cell-to-cell spread of *L. monocytogenes*	[113]
*L. monocytogenes*	SM-3, 5, 7	In vitro: on catfish fillets	Block the LapB gene, that encodes cell wall surface anchor protein	[114]
*S. aureus*, *S. epidermidis*, *S. pyogenes, S. pneumoniae* and *Bacillus cereus*	F19 and F12	In vitro on human THP-1 monocytes and mouse macrophage cell line - In mice	Host cell lysis	[115]

NI*: Not identified.

### 4.2. Quorum-Sensing/Antivirulence Inhibitors

Bacterial cells adapt to their surrounding environment and regulate their density and behavior via a cell-to-cell communication process named quorum sensing (QS). This process is mediated by bacterial secretion of extracellular signaling molecules called autoinducers (AIs) [116]. The bacteria produce and release AIs to coordinate their gene expression for survival as multicellular organisms. Additionally, AIs are also key regulators of biofilm formation, stress adaptation, secondary metabolite production, swarming motility, enzyme production, and virulence factor production [117,118]. Active transport or diffusion is used to release autoinducers into the environment to achieve efficient communication between bacterial cells [119]. As the bacterial population density rises, AIs build up in the environment, and, after this reaches a certain threshold, bacteria use them as extracellular signaling molecules to adjust their density and coordinate their gene expression [120].

QS systems are based on three fundamental concepts. (1) One is the bacterial cells’ density: at a high cell density, the cumulative generation of AIs results in local accumulation at a high concentration, which facilitates detection and response. However, at a low cell density, the AIs diffuse away, as they are present at concentrations below the detection threshold [121]. (2) Receptors generated in the cytoplasm or on the membrane are used to identify AIs. (3) The recognition of AIs leads to increased bacterial synthesis of AIs in addition to stimulation of gene expression required for cooperative behaviors [122].

Generally, the bacterial QS systems are classified into three types: (1) LuxI/LuxR–type QS, which is found in Gram-negative bacteria and uses acyl-homoserine lactones (AHL) as signaling molecules [123], (2) oligopeptide-two-component-type QS, which is found in Gram-positive bacteria and utilizes oligopeptides as signaling molecules, and (3) luxS-encoded autoinducer-2 (AI-2) QS, a general system, which is found in both Gram-negative and Gram-positive bacteria and uses AI-2 as signaling molecules [117]. The AIs are categorized into acylated homoserine lactones (AHLs), utilized by Gram-negative bacteria, oligopeptides, utilized by Gram-positive bacteria, and furanosyl borate diester, utilized by Gram-negative and Gram-positive bacteria. In addition, there are other signaling molecules of the QS system called autoinducer-3 (AI-3), which are utilized by *P. aeruginosa* and do not belong to any of the previous classes. This complex network of signals allows the bacterial community to react and adapt to different environments [124].

Interrupting the connection system between bacterial cells results in a reduction in bacterial biofilm formation and pathogenicity [124,125]. Therefore, many strategies have been developed to hinder this connection and control the QS-dependent bacterial infections [126]. The inactivation, blocking, or degradation of QS signal molecules refers to QS inhibition or quorum quenching (QQ) [26]. The perfect QS inhibitors (QSIs) are low-mass compounds with a great selectivity for the QS regulator and no deleterious side effects on the bacterium or a potential eukaryotic host. They must also be chemically stable and extremely efficient [124]. QSIs may be natural or artificial molecules. In fact, many anti-QS compounds are isolated from plants and microbes. Natural products, including plant extracts, as shown in Table 1, are the main source of QS Inhibitors (QSIs), because they contain compounds such as phenylpropanoids, flavonoids, benzoates, and gallotannins [127]. For example, grape seed extract reduces autoinducing activity and inhibits flagellum synthesis and Shiga toxin production in *E. coli* (STEC), verotoxigenic *E. coli* (VTEC), and enteroaggregative *E. coli* (EAEC) [128]. In addition, *Melia dubia* bark extracts suppress α-toxin hemolysin production, biofilm formation, and the mobility of enterohemorrhagic *E. coli* [EHEC] [129]. Furthermore, rosemary, ginger, and broccoli extracts inhibit the synthesis of AI-2 and the production of virulence factors and affect the mobility-type swarming of EHEC [130]. On the other hand, synthetic QSIs, as displayed in Table 2, include chitosan, limonene nanoemulsion, and N-phenyl-4-phenylaminothioxomenthyl amino-benzenesulfonamide, inhibiting the QS system in uropathogenic *E. coli* (UPEC), EHEC, and *S. enterica* serovar Typhimurium, respectively. The mechanisms by which QSIs inhibit QS signals include inhibition of the synthesis of AI-2 by blocking AI synthase and methyltransferase, blocking the LsrB receptor protein, inhibition of the QseC regulator protein responsible for inducing virulence factor gene expression, and inhibition of the transcriptional protein regulator LsrR or the SdiA LuxR solo regulator. Examples of QSI enzymes include AHL acylases and AHL oxidoreductase; the latter has a modified chemical structure among the AHLs [131]. The acyl side chains of the homoserine lactone are cleaved by AHL acylases, a new family of N-terminal nucleophile (NTN) that renders the AHLs activity [132]. Acyl homoserine lactone acylases have been isolated from *Streptomyces* sp. strain M664 as AhlM [133], while AHL oxidoreductase was first isolated from *Rhodococcus erythropolis* [132].

#### 4.2.1. Mechanisms of Action of QSIs

The mechanism of inhibition of QS systems includes different pathways: (1) prevention of AI synthesis [134], (2) AI receptor antagonism, (3) blocking the targets downstream of receptor binding [135], (4) application of antibodies for sequestration of AIs, (5) breakdown of AI-catalytic antibodies (abzymes) or enzymes (i.e., lactonases, acylase, and oxidoreductase) [133,136], (6) attenuation of AI secretion/transport [130], and (7) competition with autoinducing signal molecules to bind to the transcriptional protein regulators of bacterial QS systems, including *LuxS*, *LsrB*, *LsrR*, and *QscE* regulator proteins in foodborne bacteria, such as *E. coli* and *Salmonella* [136].

#### 4.2.2. Limitations of QSIs

Limitations of QSIs include (1) the development of resistance to QSIs: bacteria might evolve and develop resistance to QSIs due to the presence of plenty of QS systems in bacteria. Bacteria can regulate and induce their QS system by activating and enhancing the production of QS signal molecules, which enhance virulence factor production to promote bacterial resilience to the environment [137]. (2) Modification of virulence genes: Gram-negative bacteria can evade the action of QSIs by developing mutations in the amino acid residue of the LuxR protein regulator, which encodes virulence factors, motility, biofilm formation, and antibiotics biosynthesis [138]. (3) Indole signaling: QS systems in *E. coli* and *Salmonella* respond to indole in a nutrient-poor environment that enhances the production of virulence factors, plasmid stability, adaptation, and resistance to antibiotics. During these conditions, indole competes with AIs to bind with the AHL domain of the SdiA transcriptional regulator. Indole also competes with other QSIs that cause reduction of *P. aeruginosa*’s virulence factors, resulting in bacterial resistance from anthranilate breakdown [129,139]. (4) Disturbance of microbiota homeostasis: QSIs cause distribution of AI-2 signaling and affect human microflora activities, including adherence, biofilm formation, and production of antimicrobial metabolites, resulting in disturbance of human microbiota homeostasis [140].

**Table 2:** Quorum-sensing inhibitors with their target.

Compound Name	Source	Target Pathogen	Mechanism of Inhibition	References
C1-C10	Synthetic	APEC O78	Inhibit quorum sensing via inhibiting AI-2 production, genes associated with biofilm formation, such as the hha gene, and genes associated with bacterial cell morphology, motility, and division.	[107]
Savirin	Synthetic	*S. aureus*	Inhibits the signaling cascade of bacteria and biofilm formation by targeting AgrA to disrupt agr operon-mediated QS.	[141]
N-phenyl-4-(3-phenylthioureido) benzene sulfonamide	Synthetic	*E. coli* [EHEC]	Inhibits biofilm formation and virulence factors by modifying the AI-3 receptor (QseC).	[142]
Anti-autoinducer monoclonal antibody AP4-24H11	Synthetic	*S. aureus* [RN4850]	Inhibits the QS signaling molecule autoinducing peptide (AIP)-4 by targeting AgrA, resulting in QS inhibition and biofilm formation.	[143]
Limonene nanoemulsion	Synthetic	*E. coli* [EHEC]	Reduces AI-2 synthesis; inhibits the production of *E. coli flagellum* by inhibiting QseB and the promoter region of *flhDC* binding that encodes bacterial motility	[144]
N-phenyl-4-phenylaminothioxomen hyl amino-benzenesulfonamide	Synthetic	*E. coli* [EHEC] *S.* Typhimurium	Inhibits the histidine kinase QseC and results in a decrease in the expression of virulence factors.	[145]
Thiophene inhibitor (TF101)	Synthetic	*E. coli* (EPEC)	Inhibit the expression of the *lsrB* gene which encodes the AI-2 receptor, and interferes with *fimH,* which encodes virulence factors and inhibits biofilm formation.	[146]
Grape seed extract	Grape seed extract	*E. coli* (STEC), *E. coli* (VTEC), *E. coli* (EAEC)	Reduces the synthesis of AI and its activity by blocking AI synthase activity. Inhibits the production of *E. coli* flagellum by inhibiting QseB and the promoter region of *flhDC* binding that encodes bacterial motility and inhibit Shiga toxin production.	[128]
Thymol-carvacrol-chemotype (I and II) oils	*Lippia origanoides* *Thymus vulga0ris* oil	*E. coli* [O157:H7] *E. coli* [O33]	Inhibits the synthesis of AI-3 and prevents the formation of biofilm.	[147]
furocoumarin	Grapefruit juice	*S.* Typhimurium *E. coli* [O157:H7]	Inhibits the activity of AI-2, interferes with the activity of AI-1 molecules (AHLs), and inhibits biofilm formation.	[148]
Broccoli extracts	Basil, oregano, thyme, rosemary, ginger, and turmeric	*E. coli* [EHEC]	Inhibits the activity of AI-2 synthase and inhibits synthesis of AI-2. Affects *E. coli* mobility and inhibits production of virulence factors.	[149]
Acetic acid, citric acid, and lactic acid	Vinegar, Lemon, fermented soy products, yogurt	*S.* Typhimurium *E. coli* [O157:H7]	Inhibit the producing of the signaling molecules AI-2 by inhibiting AI-2 synthase. They also inhibit the activity of biofilm formation.	[150]
Star anise (Illicium verum Hook. f.)	Chinese fruit evergreen tree Illicium verum	*S.* Typhimurium	Interferes with promoter region *flhDC* operon which regulates the mobility. Interferes with the signal receptors *lux*, *rhl,* and *las* systems and inhibits biofilm formation.	[151]
Chitosan	Shells of crustaceans	*E. coli* [UPEC]	Reduces *E. coli* mobility by inhibiting QseB binding to the promoter region of *flhDC*. Inhibits AI-2 production and biofilm formation.	[152]
(Z)-4-Bromo-5-(bromomethylene)-3-methylfuran-2(5H)-one	Synthetic	*E. coli* [RP437]	Reduces the activity of AI-2 by reducing the activity of AI-2 synthase.	[153]
Punicalagin	Pomegranate rind	*S.* Typhimurium [SL1344]	Decreases the expression of the genes *fliA*, *fliY*, *fljB*, *flhC*, and *fimD* encoding the swimming and swarming motility of *Salmonella* and represses the expression of *sdiA* and *srgE* QS-related genes.	[154]
2,3-methyl-N-(2′-phenylethyl)-butyramide	*Halobacillus salinus*	*E. coli* [JB525]	Inhibits biofilm formation and decreases the expression of virulence factors by competing with signaling molecules (AHL) for receptor binding.	[155,156]
N-(2′-phenylethyl)-isobutyramide	*Halobacillus salinus*	*E. coli* [JB525]	Competes with signaling molecules (AHL) for receptor binding and inhibits bacterial QS resulting in biofilm formation.	[155,156]
Cyclo(L-Pro-L-Val)	*Haloterrigena hispanica*	*E. coli* [JB525]	Inhibits biofilm formation by interacting with signaling molecules (AHL).	[157]
Diketopiperazines (DKPs): Cyclo(L-Pro-L-Phe), Cyclo(L-Pro-L-Leu), Cyclo(L-Pro-L-isoLeu), Cyclo(L-Pro-D-Phe)	*Marinobacter* sp.	*E. coli* [pSB401]	Inhibits bacterial biofilm formation by inhibiting the production of AHL signaling molecules.	[158]
Kojic acid	*Altenaria* sp., from marine green algae *Ulva pertusa*	*E. coli* [pSB401]	Interferes with N-hexanoyl-L-homoserine lactone (C6-HSL) and with LuxR reporters.	[159]
*O*-prenylated flavonoid buchapine and 3-(3-methyl-2-butenyl)-4-[(3-methyl-2-butenyl) oxy]-2(1*H*)-quinolinone	*Melicope lunu-ankenda* (leaves extract)	*E. coli* [pSB401]	Inhibits biofilm formation and decreases violacein production, motility, and bioluminescence production by downregulating the expression of lecA and lux genes.	[160,161]
Sesquiterpenes, monoterpenes, hydrocarbon, and phenolic compounds. Eugenyl acetate, eugenol, and β-caryophyllene	*Syzygium aromaticum* (bud)	*E. coli* [pSB1075]	Targets lecA and lux genes resulting in the inhibition of QS-regulated phenotypes and violacein factor production, which are considered secondary metabolites responsible for growth and propagation and are a useful indicator of QS systems in bacteria.	[162]
Fructose-furoic acid	*Aloe africana* (plant extract)	*E. coli* [UPEC]	Represses the expression of biofilm phenotypic characters by competing with quorum regulator (SdiA) native ligand C8HSL.	[163]
Cembranoids	*Pseudoplexaura flagellosa* and *Eunicea knighti*	*E. coli* [pSB403] *S. aureus*	Inhibits biofilm formation by interacting with LuxR receptors.	[164]
Brominated alkaloids compounds	*Flustra foliacea*	*E. coli* [pSB403]	Inhibits biofilm formation by targeting CepR and LuxR and interferes with N-acyl-homoserine lactone.	[165]

### 4.3. Probiotics

Probiotics are microorganisms that live in a symbiotic relationship with the host. They provide health benefits and perform several biological functions when provided in adequate amounts. Probiotics were discovered and selected based on certain criteria, which ensure safety and effectiveness requirements [166]. The FAO/WHO have specified several parameters that should be assessed in vitro when selecting probiotics, such as safety, efficacy, cost effectiveness, function, and technological and physiological applications. The selected probiotics can be characterized by a lack of pathogenicity, tolerance to changes in the human gastrointestinal microenvironment, capacity for adherence to and colonization of the intestinal epithelium, antimicrobial activity, genetic and phenotypic stability, and immunomodulatory capabilities [167]. Several in vitro tests can be used to evaluate the efficacy of probiotics before starting the clinical trials, such as the agar spot test [168], the agar well diffusion assay [169], microdilution [170], antibiofilm analysis [171], 3D cell cultures, and use of human tissues and animal models [172,173,174].

Additionally, probiotics have been found to help with a variety of pathological conditions, including constipation, diarrhea, polycystic ovarian syndrome, ulcerative colitis, stress and anxiety, inflammatory bowel disease, breast cancer, and diabetes [175]. Probiotics are classified into four categories: (1) viable and active probiotics, (2) viable/non-active probiotics, in the forms of spores or vegetative cells, (3) dead/nonviable probiotics [176], and (4) next-generation probiotics [177]. The biological properties of probiotics have been extensively investigated, but only a few studies focused on their antimicrobial properties as novel antibiotic alternatives.

1. Viable and active probiotics

**Figure 3:**
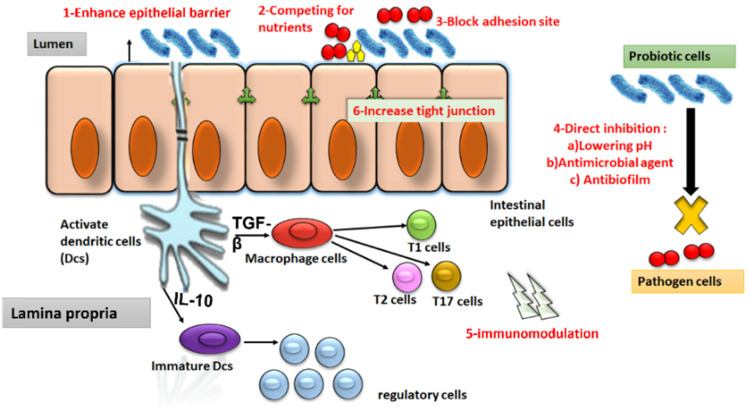
Mechanism of probiotics action.

According to the FAO/WHO, probiotics delivered into the body via the gastrointestinal tract (GIT) need to be viable and active in vitro (externally), resistant to GIT conditions, and viable and active in vivo (internally) [178]. Viable and active probiotics provide health benefits to the host via (1) increasing the hydrogen ion concentration (low pH value) in the gut, (2) enhancing synthesis of essential vitamins and enzymes, (3) production of antimicrobial substances, (4) restoring intestinal microbiota after diarrhea, (5) lowering serum cholesterol, (6) boosting the immune system, (7) production of antioxidants, (8) reduction of food allergy sensitivities, and (9) increasing lactose and calcium absorption [179]. Probiotics should be alive when traveling from the mouth to the gut and resist saliva enzymes, gastric fluid (acid and enzymes), bile salts, competitive gut microbiota, and inhibitory GIT conditions. Lactic acid bacteria (LAB) are considered the major probiotic bacteria that are used as viable cells, including homofermentative lactobacilli, which are represented by three groups, (i) the *L. acidophilus* group (*L. acidophilus*, *L. johnsonii*, *L. crispatus* and *L. gasseri*), (ii) the *L. salivarius* group, and (iii) the *L. casei* group (*L. paracasei*, *L. zeae* and *L. rhamnosus*). Additionally, Reuter and coworkers reported that *L. fermentum* is the predominant heterofermentative lactobacilli associated with the human GIT [180]. Other LABs that have been isolated either from dairy products or human GIT are *Bifidobacterium animalis*, *B. bifidum*, *B. breves*, *B. infantis*, *B. lactis*, *B. longum*, and *E. faecium*. In addition, non-lactic acid producers include *B. cereus*, *E. coli* Nissle 1917, *Sporolactobacillus inulinus*, *Propionibacterium freudenreichii*, and *Saccharomyces cerevisiae* [181]. Applications of selected probiotics against pathogenic bacteria and their mechanisms of action are summarized in Table 3 and Figure 3.

2. Viable and inactive probiotics

These are viable probiotics but are not metabolically active. They are considered dormant probiotics because they are exposed to detrimental stresses such as temperature, extreme pH values, high osmotic pressure, and high O_2_ supplementation (for anaerobes) [182,183]. *Bacillus* species, such as *B. coagulans*, *B. subtilis*, *B. clausii*, and *B. licheniformis*, have lately been authorized as inactive viable probiotics and utilized in human diets as well as for the treatment of intestinal and urinary problems [184]. Bacteriocins are produced by *Bacillus* spp. and are effective against Gram-positive and Gram-negative bacteria and fungus found in the food. As a result, they are commonly used in the food industry as natural preservatives [185]. *B. clausii* have been reported to treat child diarrhea, allergic children’s immune systems, respiratory infections, and *Helicobacter pylori* infections (Table 3) [186].

3. Dead/nonviable probiotics (postbiotics/parabiotics)

The host can be protected from harmful microbes by using dead/nonviable probiotic cells [176]. Different procedures are performed for obtaining nonviable/inviable/inactivated/dead probiotic cells, including exposure to ultraviolet (UV) radiation for 530 min, heat at 121 °C for 560 min, and ionizing radiation (10 kGy). Protein denaturation, enzyme inactivation, nucleotide destruction, DNA breakage, and cell structural deformation are among the structural and functional changes associated with the inactivation pathway. This is called parabiotic/postbiotics, and is considered a new horizon in microbial therapy and the food industry [187]. In mice, heat-inactivated *L. plantarum* has prevented *S. enterica* infection in multiple organs, including the liver, spleen, and blood, by reducing pathogenic cell translocation and adhesion into intestinal cells [188]. Through enhancing the host immune responses (local and systemic), heat-inactivated *Leuconostoc mesenteroides* cells prevented *L. monocytogenes* invasion into Caco-2 cells [189]. Along similar lines, inactivated heat-killed yogurt prevent cytokine-induced barrier disruption in human intestinal epithelial cells [190]. Inactivated *L. paracasei* and *L. rhamnosus* cells have been found to prevent colon and stomach cancer by reducing proliferative activity and improving cancer cell apoptosis [191]. They can also lessen allergic rhinitis symptoms by maintaining cell wall integrity [192]. In hamsters with allergic rhinitis, administration of heat-inactivated *E. faecalis* FK-23 cells increased the number of T-regulatory cells in the spleen and altered the body’s immune responses [193]. Heat-killed *L. rhamnosus* CNCM-I-3698 and *L. farciminis* CNCM-I-3699 exhibited coaggregation potential against foodborne pathogens, such as *Campylobacter*, *Salmonella*, *E. coli*, and *L. monocytogenes* [194]. It is noteworthy that the strongest coaggregation was mediated by a carbohydrate–lectin interaction between the heat-killed strains and *C. jejuni* CIP 70.2 and resulted in inhibition of its attachment to intestinal tissues [194]. Additionally, *L. brevis* cells have been shown to suppress the transcription of tumor necrosis factor, reduce the expression of sterol regulatory element binding protein 1 and 2, and enhance the induction of heat-shocked protein in the gut [195]. Lipoteichoic acid (LTA) is a microbe-associated molecular pattern (MAMP) expressed by Gram-positive bacteria and detected by the Toll-like receptor 2 (TLR-2) expressed on the surface of gut enterocytes. Ligation of LTA to TLR-2 initiates cellular signals, leading to induction of an inflammatory cytokine response. LTA derived from probiotic *Lactobacillus* strains has anti-biofilm properties against oral and enteric pathogens, such as *S. mutans*, *S. aureus*, and *E. faecalis,* by inhibiting biofilm formation and destroying pre-existing biofilms [187]. Parabiotics and postbiotics have several advantages over live probiotics, including easier creation and storage, possession of particular mechanisms of action, better accessibility of MAMP during contact with pattern recognition receptors (PRR), and greater likelihood of inducing targeted reactions through specific ligand–receptor interactions [187].

4. Next-generation and genetically modified probiotics

Next-generation probiotics (NGP) are those commensal and typical occupants of GIT microbial strains that enhance host defense against gastrointestinal pathogens [196]. Most NGP are gut bacteria that are nutritionally finicky and oxygen-sensitive, such as genera *Bacteroides*, *Clostridium*, *Faecalibacterium*, and *Akkermansia*, as well as genetically engineered (GE) strains [177]. As such, they are difficult to mass-produce and keep alive during processing and eventual product formulation [197]. They also require careful target consumer selection and circumstances; unlike other conventional probiotics, they are not suited to or safe for all users [196]. For example, *Bacteroides* spp. are essential gut microbiota members with a high capacity to metabolize complex polysaccharides that can help other bacteria. *Bacteroide* spp. such as *B. thetaiotamicron* have been found to interact with intestinal cells and modulate the expression of the host genes by inducing dendritic cell immunotolerance [198,199]. With its zwitterionic polysaccharide, *B. fragilis* can stimulate the host immune system. *C. butyricum* is a spore-forming, Gram-positive, butyrate-producing anaerobe of human and animal guts. While butyrate is known to exert beneficial effects on the host, supplementation of *C. butyricum* to newborns can induce necrotizing enterocolitis and type *E botulism* [198,199]. *Faecalibacterium prausnitzii* is a non-spore-forming, Gram-negative, butyrate-producing anaerobe (extremely oxygen-sensitive, EOS). The lower abundance or absence of *F. prausnitzii* has been linked to a variety of gastrointestinal illnesses [200]. This species is technologically problematic because of its great sensitivity to oxygen. Many experiments with *F. prausnitzii* have used culture supernatants (SN) instead of live cells to circumvent concerns with viability and stability [177]. The butyrate synthesis and immunomodulatory activities of *F. prausnitzii* have been related to its potential health benefits [177]. Additionally, *F. prausnitzii* (live cells or SN) was found to prevent colitis caused by dinitrobenzene sulfonic acid (DNBS), trinitrobenzene sulfonic acid (TNBS), or dextran sodium sulfate (DSS) (doses 10^9^ CFU/day) [201]. Live cells of *F. prausnitzii* were proven to reduce the incidence of diarrhea and mortality-associated diarrhea in dairy calves and increase body weight [202].

#### 4.3.1. Mechanisms of Action of Probiotics

Probiotics exert their effects via (1) the sustainability of host–microbe interactions and pathogen prevention through competitive exclusion. Probiotics competitively exclude pathogens by a variety of mechanisms, including competing with them for nutrients, blocking the epithelial adhesion sites, and decreasing the intestinal lumen pH [203]. (2) Production of antibacterial compounds. Compounds that are produced in the metabolome of probiotics include organic acids (butyric, lactic, and acetic acids), bacteriocins, hydrogen peroxide, amines and peptides, which not only antagonize opportunistic pathogens but also play a crucial role in regulation of host cellular proliferation, differentiation, inflammation, angiogenesis, and metastasis [204]. The mechanisms by which probiotics antagonize microbial growth include a reduction in intestinal pH [205], pathogen agglutination, toxic substance entrapment and metabolization [206], alteration of the intestine’s motility [207], and production of bacteriocins, H_2_O_2_, and organic acids (Figure 3) [176]. (3) Promoting the synthesis and secretion of mucus by intestinal goblet cells to form a protective layer against pathogens [208]. (4) Stimulation of the host immune system [178,209]. (5) Production of vitamins, minerals and trace elements and important digestive enzymes (e.g., b-galactosidase) [210]. (6) Inhibition of the adherence and colonization of opportunistic and pathogenic bacteria. (7) Enhancing and maintaining gut mucosal integrity.

#### 4.3.2. Limitations of Probiotics

Despite variable scientific evidence reporting the beneficial health impact of probiotics, concerns continue to grow about their clinical applications due to some obstacles, including: (i) the loss of viability of true probiotics during the preservation period, particularly at room temperature [211], (ii) different colonization patterns and variable tolerance to gut conditions, (iii) the potential of acquisition of virulence genes from opportunistic or pathogenic organisms, (iv) the capacity of some probiotic strains to transfer antibiotic resistance genes within the GIT [212], and (v) the probability of production of toxic substances, such as the heat-stable amylosin toxin from *Bacillus amyloliquefaeiens*, *B. subtilis*, and *B. mojavensis*, which may induce food poisoning [213].

**Table 3:** Applications of selected probiotics against pathogenic bacteria and their mode of action.

Probiotic	Target Pathogen	Additional Benefits	Monitoring	References
Nissle *E. coli* 1917 (EcN)	*C. jejuni*	In vitro: enhance tight junction functions and modulate the innate immune response on HT-29 cells. In chickens: reduce *C. Jejuni* colonization in the cecum up to 2.5 logs; enhance the immune response and intestinal morphology of the treated chickens without showing adverse effect on the gut microbe.	In vitro (HT-29 cell line) In chickens	[172,173,174,214]
*L. plantarum*	*L. monocytogenes*, *S.* Enteritidis, *E. coli* O157:H7 and *Staphylococcus*	Attach to epithelial cells, stimulate the production of IL-10 in the colon, and enhance the induction of dopamine and serotonin.	In vitro & in mice	[188,215]
*L. paracasei & L. rhamnosus*	*E. coli* V517, *S.* Enteritidis *OMS-Ca*, *S. aureus* 76 and *L. monocytogenes* ATCC 15313	Boost mineral bioavailability in food products, reduce serum parathyroid hormone via synthesis of short-chain fatty acids, enhance mineral solubilization and absorption, production of phytase, and hydrolyze glycoside linkages of estrogenic food products.	In vitro	[191,216,217]
*L. helveticus*	*L. monocytogenes* ATCC 19115, *S.* Typhimurium ATCC 14028, *S. aureus* ATCC 25923, and *E. coli* O157:H7 ATCC 43889	Stop GIT infections, improve protection against pathogens, enhance the immune system of the host, and makeup the composition GIT microbiota.	In vitro	[218,219]
*L. reuteri*	*E. coli* ATCC25922, *S. typhi* NCDC113, *L. monocytogenes* ATCC53135, and *E. faecalis* NCDC115.	Reduce pro-inflammatory cytokines production, promote regulatory T cells, strengthen the intestinal barrier, and decrease microbial translocation from the gut lumen to the tissues.	In vivo	[220,221]
*L. acidophilus*	*S. aureus*, *P. aeruginosa*, *L. monocytogenes*, *V. parahaemolyticus*, *V. cholerae*, *H. pylori*, *Klebsiella*, *Salmonella*, *Shigella*, *Bacillus*, *Clostridium*, *Mucor*, *Aspergillus*, *Fusarium*, *Trichoderma* and *Candida* spp.	Production of lactacins B and F, acidophilin, acidocin, acidophilucin, and acidophilicin.	In vitro	[222]
*L. rhamnosus* GG and *B. lactis* Bb12	APEC	Reduce the number of colonized APEC in chicken cecum with modulation of the gut microbiota.	In vitro In chickens	[223]
*S. lactis* and *L. delbrueckii subsp. Bulgaricus*	*E. coli* ATCC25922 and *S. aureus* ATCC25923	Inhibit proliferation via production of acid metabolites.	In vitro & in vivo	[224]
*B. animalis* AHC7	*S.* Typhimurium	Mediate weakness of activation of NF-κB that includes recognition of the pathogen by dendritic cells and production of T cells.	In humans	[225]
*B. adolescentis and B. pseudocatenulatum*, *and B. longum*	Vancomycin-resistant *S. aureus* and *Enterococcus*, *Propionibacterium acnes*, *S. aureus,* and *S. Epidermidis*	Reduce pathogen growth and cell adhesion.	In vitro	[226]
*B. bifidum and B.m infantis*	*S. enterica* serotype Enteritidis	Reduce pathogen growth via production of acids, hydrogen proxide, and bacteriocins.	In vitro	[227]
*B. lactis*	*S.* Typhimurium	Stimulate transient pro-inflammatory host responses in the epithelial cells of the intestine.	In vivo (rats)	[228]
*Propionibacterium freudenreichii*	Multidrug-resistant *S.* Heidelberg	Anti-inflammatory effect.	In vitro (HT-29 cell line)	[229,230]
*Pediococcus acidilactici* Kp1	*L. monocytgenes*, *S. enterica*, *Shigella sonnei*, *Klebsiella oxytoca*, *Enterobacter cloaca* and *S. pyogenes*.	Hender the adherence of pathogens to the intestinal mucosa by forming a barrier via auto-aggregation; production of bacteriocin-like inhibitory substances.	In vitro	[231]
*Leuconostoc mesenteroides*	*L. innocua*, *L. ivanovii*, or *S. aureus*	Production of bacteriocin, which inhibits the growth of pathogens, and lowering the medium pH.	In vivo (mice)	[189,232]
*E. faecium* NCIMB 11181	*C. perfringens*	Ameliorate necrotic enteritis and reduce intestinal barrier injury.	In chickens)	[233]
*S. salivarius* K12	*S. mutans* and *S. hominis*	Antibiofilm of *Schaalia odontolytica* P10 and *Enterobacter cloacae.*	In vitro	[234]
*S. thermophilus* SMQ-301	*S. aureus*, *E. coli*, and *Gardnerella vaginalis*	Potential candidate for novel biotherapeutic interventions against inflammation caused in septic mice.	In vitro, in vivo	[235,236]
*B. coagulans subtilis*, *B. laterosporus*	*E. coli*, *P. aeruginosa*, *K. pneumoniae*, *B. subtilis*, *S. aureu*, and *Candida albicans*	Stimulate human immune cells and change the induction of anti-inflammatory cytokines and chemokines.	In vitro (cell lines)	[237]
*Saccharomyces boulardii*	*S. aureus*, *E. coli*, *Klebsiella oxytoca*, *Yersinia enterocolitica*, *C. perfringens*, *C. difficile*, *Salmonella* sp., *Shigella* sp., *Candida albicans* and *Entamoeba hystolitica*	Affect the epithelial reconstitution; anti-secretory, anti-inflammatory, and immunomodulating effects.	In vivo (Lymphocyte-transferred SCID mice)	[238,239,240]
*C. butyricum* (CBM 588)	*E. coli* [EHEC] O157:H7	Inhibit growth by limiting the adhesion of pathogen to epithelial cells and the production of butyric acid.	In vivo (mice)	[241,242]
*L. salivarius*, *L. johnsonii*, *L. reuteri*, *L. crispatus*, and *L. gasseri*	*C. jejuni* 81-176	Inhibit the quorum-sensing signals of *C. jejuni.* Reduce the expression of *C. jejuni* virulence-related genes, including genes responsible for motility (*flaA, flaB*, and *flhA*), invasion (*ciaB*), and AI-2 production (*luxS*). Enhance the phagocytic activity of macrophages. Increase the expression of cytokines and co-stimulatory molecules in macrophages.	In vitro	[243]
Microbial consortia (Aviguard and CEL)	*C. jejuni* 81-176	Enhance the intestinal mucosa via the modulation of gut microbiome composition by increasing the relative abundance of Bacteroidaceae and Rikenellaceae	In vivo (chicken)	[244]
*L. johnsonii*, *Ligilactobacillus salivarius*, *Limosilactobacillus reuteri*, and *L. crispatus*	*C. perferingens*	Induce significant alterations in cytokine gene expression in the intestine. Modify the gut microbiome composition. Improve intestinal morphology.	In vivo (chicken)	[245]

### 4.4. Prebiotics

Prebiotics are defined as “non-digestible food materials that beneficially impact the host by selectively enhancing the growth and/or metabolism of bacterial species inhabiting the GIT, and thus improve the host health” [246]. Prebiotics are also defined as “any substrate preferentially consumed by host microorganisms that result in increasing the health benefit” [246]. Evidence indicates that prebiotics are promising alternatives in the medicinal and food industries. Prebiotics are characterized by (1) the ability to withstand the acidic environment during passage through the digestive tract (GIT) [247], (2) resistance to digestive enzymes but susceptibility to probiotic-hydrolyzing enzymes [248,249], (3) non-direct absorbance [250], (4) maintenance of gut microbial ecology [248], and (5) the ability to stimulate the host immune response [247]. Prebiotics are non-digestible oligosaccharides (fructans, inulins, xylans, galactans, and mannan), fibers (pectin, non-starch polysaccharides (such as β-glucan), xylooligosaccharides, andisomaltooligosaccharide), and seeds containing gums [251,252]. Human milk oligosaccharide is considered an endogenous source of prebiotics that increases the population of *Bifidobacterium* spp. in breastfeeding newborns, thereby enhancing their immunity [253]. To use prebiotics as alternatives to antibiotics, specific criteria must be met. Prebiotics should have a well-identified source chemical composition and structure, be a pure product, be at a suitable dose, and have been assessed in animal models or 3D cells to confirm their safety and beneficial health impact on the microflora [252]. When used as feed additives for livestock and poultry, prebiotics have shown an ability to improve host health and productivity via selective stimulation of proliferation and metabolism of the gut microbiota, such as *Akkermansia* spp., *Christensenella* spp., *Propionibacterium* spp., *Faecalibacterium* spp. and *Roseburia* spp., *Lactobacillus* spp., *and Bifidobacterium* spp. [254]. In the context of their benefits for human health, fermentation of the prebiotics konjac glucomannan hydrolysate and inulin in a batch culture of human feces has been associated with the production of short-chain fatty acids and proliferation of the genera *Bifdobacterium*, *Lactobacillus* and *Enterococcus* [255]. A meta-analysis of 64 studies reported that addition of dietary fibers stimulated *Bifidobacterium* spp. and *Lactobacillus* spp. resulting in an increase in the concentration of fecal short-chain fatty acids (SCFAs) in healthy adults [256]. Moreover, other studies have revealed that these bacteria play a key role in maintaining the composition of GIT microflora, enhancing the food and mineral absorption, and promoting the host defense system [257].

Prebiotics have also shown potential to eliminate harmful bacteria, such as *Salmonella*, *Campylobacter*, *Clostridium* and *E. coli* [258,259]; however, their mechanism of action remains to be elucidated. Fermentation of prebiotics by the gut’s resident microbiota and the subsequent production of SCFAs results in a reduction in the gut pH, which, in turn, make the condition unfavorable for the growth and colonization of invading pathogens [260]. It was reported that the activity of probiotic *Bifidobacterium* strains against *C. difficile* was significantly stimulated in the presence of five prebiotics (oligosaccharides) [261]. Similarly, the activity of *Pediococcus acidilactici* was enhanced against *E. coli, Salmonella*, *E. fecalis* and *S. aureus* in the presence of garlic and basil (natural prebiotics) [262]. Moreover, the use of the prebiotics mannan-oligosaccharides and fructooligosaccharides, as poultry feed supplements, reduced the colonization of *Campylobacter* and *Salmonella* in the GIT of poultry [259]. Supplementation of weaning pigs with prebiotic oligofructose resulted in a significant increase in the number of *Bifidobacteria* and *Lactobacilli* and a significant reduction in the number of clostridia, enterobacteria, and enterococci [263]. A reduction in disease severity was observed following treatment of patients with *C. difficile*-associated diarrhea with inulin and oligofructose [264].

#### 4.4.1. Mechanisms of Action of Prebiotics

There is no distinctive mechanism by which prebiotics eliminate the pathogenic bacteria, stimulate the host GIT microflora, or enhance immunity. Indeed, the mechanisms of action of prebiotics are very complex, as they are associated with a set of actions on the host physiology and intestinal microflora balance [252],. Nonetheless, it is well documented that fermentation or degradation of prebiotics beneficially affect the host by (1) mediating the growth and proliferation of beneficial intestinal microbes [260], (2) blocking adhesion sites on the epithelial cells (the catabolic end products released from the bifidogenic degradation of prebiotics might block the adhesion sites on the epithelial cells), (3) acting as receptor analogues and blocking lectin receptors that present on the surface of the pathogens [265], and (4) producing SCFAs, such as acetate, propionate, lactate, and butyrate, which result in lowering of the pH of the intestine, leading to suppression of the growth and colonization of pathogenic bacteria [266]. Additionally, SCFAs serve as a source of energy for the intestinal cells, in addition to their role in maintaining intestinal integrity by enhancing the expression of the tight junction proteins [267].

#### 4.4.2. Limitations of Prebiotics

Prebiotics lack life-threatening or severe side effects. However, there are a few moderate side effects that have been reported [249], including osmotic diarrhea, bloating, cramping, and flatulence. It should be noted that these side effects depend upon the chemical composition of the prebiotics and the length of side chain, with short side chains reported to have more side effects than long ones [268]. Additionally, prebiotic dose is a critical factor that affects their safety profile. The commercially recommended dose of prebiotics is 1.5–5 g per portion [268].

### 4.5. Antimicrobial Peptides (AMPs)

AMPs are naturally produced by various immune cells and play a vital role in the innate immune systems of various animals, plants, and microorganisms [269]. AMPs, have a wide spectrum of antimicrobial activity against bacteria, fungi, viruses, and parasites [270]. In addition to their antimicrobial activities, AMPs possess biological functions, such as immune modulation, angiogenesis, antitumor activity, and wound healing [271,272]. AMPs are considered promising alternatives to antibiotics, due to many advantages; (1) they act on multiple target sites on the intracellular targets and plasma membranes of pathogenic bacteria, (2) they have potent killing activity against drug-resistant bacteria [270,273], (3) they are a component of the innate immune system, (4) their natural production by the host cells saves time and energy compared to antibody synthesis via acquired immunity, and (5) they reach the target sites faster than immunoglobulin [269]. AMPs are classified into several subgroups based on their amino acid sequences (10–100), the net charge of the peptide (+2 to +9), and their protein structure and sources [274]. These subgroups include (1) anionic AMPs, which consist of 5–70 amino acid residues and have a net charge range of −1 to −8 [275]. Their structural characteristics include α-helical peptides and cyclic cystine knots [275]. They use the negatively charged content of the microbial membrane to form salt bridges, leading to disruption of the microbial membrane [276]. (2) Cationic α-helical AMPs are ≤40 amino acids in length (50% hydrophobic in nature) and have a charge of +2 to +9 and the C-terminus amidated [277]. The structure of cationic α-helical AMPs is disordered in aqueous solutions [278]. They are capable of forming amphiphilic structures when interacting with target cells [279]. (3) Cationic AMPs, where the peptides consist of 2–8 cysteine residues forming 1–4 pairs of intramolecular disulfide bonds. These disulfide bonds play a crucial role in β-sheet AMP stabilization and biological functions [280]. (4) Extended cationic AMPs containing amino acids including tryptophan, arginine, proline, histidine and glycine and lacks the regular secondary structures [279]. (5) Fragments from antimicrobial proteins that have a broad-spectrum bactericidal effect, such as lysozyme [281]. The helix–loop–helix (HLH) region in the human and chicken lysozyme has a strong effect on the growth of pathogenic bacteria and fungi [282].

Many antimicrobial peptides, isolated from different sources, have shown activity against a wide variety of pathogenic bacteria. For example, magainin-2 (α-helix (TFE)) was originally isolated from frogs and has been shown to be active against *P*. *gingivalis*, *F. nucleatum*, *P. intermedia*, *E. coli*, and *S. aureus* [283]. Cecropin and cecropin P1 (α-helix structure) were isolated from silk moth and pig, respectively. These peptides inhibited the growth of *E. coli* ML-35p [284], *S. aureus*, *B. subtilis*, *M. luteus*, *P. aeruginosa*, *S.* Typhimurium, *S. marcescens* and *E. coli* [285]. In addition, apo-lactoferrin (α-helix structure), discovered in bovine and human PDB code (1BOL), inhibited the growth of *E. coli* O157:H7 [286]. Melittin (α-helix structure), extracted from bee, was active against *S. salivarius*, *S. mitis*, *S. mutans*, *S. sanguinis*, *S. sobrinus*, *L. casei*, and *E. faecalis* [287]. Temporin A and temporin L, extracted from frog, were active against MRSA [288], *B. megaterium* Bm11, *S. aureus* Cowan I, and *E.coli* D21 [289]. Moreover, buforin II and clavanin A (α-helix structure), discovered in extended toad and Styela clava, respectively, possess inhibitory activity against *B. subtilis*, *S. aureus*, *E. coli*, *S.* Typhimurium [290], *E. coli* ML35p and *L. monocytogenes* [291]. Furthermore, protegrin-1 (β-sheet structure), isolated from human and porcine, has demonstrated antagonistic activity against MRSA and *P. aeruginosa* [292]. Tachyplesin-I (β-sheet structure), discovered in horseshoe crab, was shown to inhibit the growth of *S. Typhimurium* [293]. Furthermore, hepcidin (β-structure), extracted from humans, has demonstrated capabilities to inhibit *E. coli, S. aureus*, and *S. epidermidis* [294]. Daptomycin (cyclic lipopeptide membrane), isolated from *Streptomyces roseosporus*, can kill MRSA [295] and nisin (lantibiotic), isolated from *L. lactis*, was shown to kill MRSA, *S. pneumoniae*, *Enterococci* and *C. difficile* [296]. NPSRQERR [P1], PDENK [P2], and VHTAPK [P3]), derived from *L. rhamnosus* GG, showed inhibitory activity against APEC in chicken [297].

#### 4.5.1. Mechanisms of Action of AMPs

**Figure 4:**
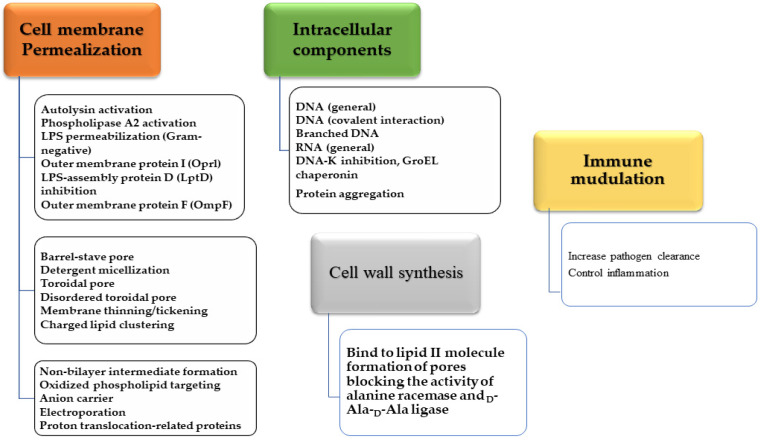
The mechanisms of antibacterial action of AMPs.

The antibacterial activity of AMPs depends mainly on the type and the nature of the AMPs as well as the targeted bacterial pathogens. AMPs exert their antibacterial activity through either direct killing mechanisms where they cause membrane disruption, eventually causing bacterial cell death, and/or indirect mechanisma via modulation of the immune system (Figure 4) [298,299]. Cationic AMPs exert antibacterial activity by interacting with negatively charged bacterial membranes, leading to increased membrane permeability and cell lysis [269]. α-helical AMPs can bind to an anionic lipid microbial membrane and modify its disordered structure in aqueous solution into an amphiphilic α-helical structure to enhance its interaction with the microbial membrane [300]. Other modes of action of cationic AMPs were found to be dependent upon the pH of the medium. At neutral pH, AMP (clavanin A) adopts the membrane permeation mode of α-helical peptides [301], while at a slightly acidic pH, it induces cell death by disturbing membrane proteins. Additionally, AMPs such as buforin II, indolicidin, and Peptide-P2 [302] can pass through the bacterial membranes and bind to DNA, inhibiting enzyme synthesis and indirectly stopping DNA replication and transcription [303]. Some AMPs, such as PR-39, a proline- and arginine-rich AMP, oncocin-type peptide, and Api137, an apidaecin-type peptide, inhibit protein synthesis via inhibition of mRNA translation, blocking the assembly of the ribosome 50S large subunit, or binding to the tunnel of ribosomes and preventing the transition from the initial stage to the elongation stage of translation [304]. Microcin J25 was found to bind to the secondary channel of the RNA polymerase and block trigger-loop folding. LL-37 was shown to inhibit *E. coli* via stopping the activity of palmitoyl transferase PagP [305]. NP-6 was found to inhibit the β-galactosidase activity of *E. coli* [306]. Further, cycloserine was also found to inhibit bacterial cell wall biosynthesis via blocking of the activity of alanine racemase and _D_-Ala-_D_-Ala ligase and, consequently, the synthesis of the _D_-Ala-_D_-Ala dipeptide of lipid II of the peptidoglycan precursor [269]. This suggests that HNP1 kills bacteria by interacting with lipid II. Teixobactin inhibits the synthesis of bacterial cell walls by binding to a highly conserved motif of lipid II and lipid III (precursors of cell wall teichoic acid) [298]. Amyloidogenic peptide and amyloids cause direct protein coaggregation leading to suppression of intracellular transport processes and eventual bacterial death [307].

#### 4.5.2. Limitation of AMPs

Although large numbers of AMPs have been discovered and characterized, a small number have been applied in clinical trials and a limited number have been approved by the FDA [298]. Most clinically applied AMPs are limited to topical applications, due to their systemic toxicity, the susceptibility of the peptides to protease degradation, and rapid kidney clearance if they are administrated orally [308]. Additionally, oral administration of AMPs can lead to proteolytic digestion by GIT enzymes, such as trypsin and pepsin, while systemic administration leads to a short half-life, protease degradation, and cytotoxic profiles in blood [309].

### 4.6. Bacteriophages

Phages or bacterial viruses are obligate parasites that infect bacteria and archaea. Phages are classified according to their size, shape, type of nucleic acid and mechanism of action in the host bacterial cell [310]. The genomic sequences of phages range from a few thousand base pairs to 498 kilobase pairs in phage G, the biggest phage ever sequenced [311]. Some phages have wide host ranges; however, the majority of them have high host specificity [312]. Based on the last classification, bacteriophages are classified as virulent (e.g., lysis of the bacterial cells to release new phages) or temperate (e.g., incorporation of its genetic material in the host genome and the host changes its genetic characters) bacteriophages [310]. In vitro trials showed that phages have many advantages over antibiotics, including (1) high host specificity (phages can target one strain of bacteria without perturbing the human or animal gut microbiota, while antibiotics do not distinguish between pathogenic and beneficial bacteria); (2) cost effectiveness and time saving [313]; (3) inhibition of multi-drug-resistant bacteria while antibiotics increase them; (4) ease of delivery to the target site and ability to penetrate the blood–brain barrier [314]; (5) no antagonistic effect detected between phages when given as a cocktail (mixture of different phages); (6) that phages could prevent biofilm formation [315]; and (7) phages might be used as an alternative in antibiotic-allergic patients; however, very few reports discussed this [316]. Several studies have reported that phage therapy development might be a potential solution to bacterial antibiotic resistance as well as the treatment of numerous bacterial infectious illnesses [317]. As the poultry gut is considered the main reservoir of *Campylobacter*, most *Campylobacter* phages have been isolated from avian GIT. Many studies have demonstrated that administration of individual phages resulted in a significant decrease in the number of *Campylobacter* without altering the GIT microbiota [318]. The most multi-drug-resistant bacterial strains, such as *E. faecium*, *S. aureus*, *K. pneumoniae*, *A. baumannii*, *P. aeruginosa*, and *Enterobacter* spp, have been reported to cause serious human diseases. The specific types of phages that have been applied for treatment of these pathogens are summarized in Table 4.

#### 4.6.1. Mechanisms of Action of Bacteriophages

The majority of phages are virulent (lytic) with a negligible probability of becoming temperate (lysogenic) [314]. The lytic cycle of virulent phages starts by attachment of tailed phage to the cell surface receptors of a host. This interaction is two steps, a reversible phase followed by an irreversible phase. After the irreversible attachment, the lysis enzymes break down the host cell wall and the phage ejects its genetic material into the host cell with the assistance of processive host enzymes. The phage genome then manipulates the host cell metabolism by redirecting it for DNA replication and protein biosynthesis to the production of phage particles (nucleic acids and capsids); the host cell genome is degraded at this stage, followed by phage particle assembly and host cell lysis. The new viral particles are then released to re-attach to a new host cell. The lytic cycle of bacteriophages is performed by key phage proteins (holins) that permeabilize the host cell membrane and endolysins that degrade cell wall peptidoglycan. Consequently, the bacterial cells lose their cell wall integrity and the selective permeability of cell membranes, eventually resulting in cell lysis due to osmotic disruption [319].

#### 4.6.2. Limitations of Phage Therapy

The main limitations for application of phage therapy are: (1) high host specificity. This can be overcome by using a cocktail of phages that may kill a broader host range. However, this is only feasible in chronic infection, not acute infection. Thus, a stable cocktail and/or phage lytic enzymes are recommended as alternatives [312]. (2) The targeted bacteria may develop resistance over time against phage attachment and adsorption by altering the receptor sites [312]. (3) Some bacteria produce endotoxins during lysis by phages, which may lead to septic shock. However, it should be noted that induction of endotoxins by antibiotic treatments like amikacin, cefoxitin, and imipenem was reported to be higher than that released from coliphage treatments [320]. (4) It is difficult to obtain regulatory approval for phage-based therapeutics in in vivo trials [317]. (5) It is difficult to control the stability and purity of phages that are prepared for clinical trials, which may result in low-quality control data [321]. (6) Temperate phages are not preferable for therapeutic applications, due to the fact that phage-induced changes in the host DNA may lead to spread of antibiotic resistance [319,322]. (7) There is a significant decrease in phage concentrations by the reticuloendothelial system or neutralization by antibodies during therapeutic application [323].

**Table 4:** Examples of identified phages against pathogenic bacteria.

Phage	Target Bacteria	PFU	Application	Reference
Cocktail of 12 natural virulent bacteriophages	*P. aeruginosa*	10^6^	In vivo in human	[324]
coliphage PhiX174	*S. aureus*	NI	Patients with *S. aureus* bacteremia	[325]
Phage cocktail DS-6A, GR-21/T, My-327	*Mycobacterium abscessus*	10^9^	A cystic fibrosis patient	[326]
cocktail 1 (*P. aeruginosa* 24, *P. aeruginosa* 25, and *P. aeruginosa* 7)	*P. aeruginosa*	6.2 × 10^10^	Mice with chronic bacterial lung infections	[327]
IME-AB2	*A. baumannii*	62 PFU/cell	Reduce lung inflammation in mice	[328]
Pyo phage phage cocktail from the Eliava Institute	*S. aureus*, *E. coli*, *Streptococcus*, *P. aeruginosa*, or *Proteus mirabilis*	10^7^–10^9^	Patients with urinary tract infections	[329]
T4-like coliphage cocktail	*E. coli*	3.6 × 10^8^	Diarrhea infected children	[330]
WPP-201 phage coctail	*P. aeruginosa*, *S. aureus*, and *E. coli*	8 × 10^7^	Leg ulcer patients	[331]
*P*. *aeruginosa* phages 14/1 (Myoviridae) and PNM (Podoviridae) and *S. aureus* phage ISP (Myoviridae),	*P. aeruginosa* and *S. aureus*	10^9^	Colonized burn wounds	[332]
PP01 phage,	*E. coli* O157: H7	10^5^	In vitro	[333]
PlySs2 and PlySs9	*S. uberis*	NI	In vitro (bovine mastitis)	[334]
PlySs2	*S. equi*, *S. agalactiae*, *S. dysgalactiae*, *S. pyogenes*, *S. sanguinis*, *S. pneumoniae* and *group E streptococci*	NI	In vitro and in vivo (mice)	[335]
Φ7-izsam and Φ16-izsam	*C. jejuni*	10^7^	In chickens	[336]
Phage cocktail e11/2, e4/1c, pp01	*E. coli* O157:H7	ND	Meat surface	[337]
Phage Cj6	*C. jejuni*	5 × 10^8^	Raw and cooked beef	[338]
Phage Φ2	*C. jejuni*	10^7^	Chicken skin	[339]
*Salmonella* phage (P7)	*Salmonella*	5 × 10^8^	Raw and cooked beef	[338]
phage SJ2	*S.* Enteritidis	104	Cheddar cheese made from raw and pasteurized milk	[340]
phage A511	*L. monocytogenes*	5.2 × 10⁷	Red smear cheese	[341]
Cocktail of the two lytic phages	*S. aureus*	10^6^	Fresh and hard cheese type	[342]

PFU; plaque-forming units per mL, NI; Not identified.

### 4.7. Nanoparticles (NPs)

NPs are considered one of the potential alternative candidates of antibiotics for controlling multi-drug-resistant microorganisms [343]. They have demonstrated therapeutic potential due to their unique chemical and physical characteristics [344]. NPs have a tiny size (1–100 nm) with a large surface area to interact with target organisms [345]. They can be chemically or naturally synthesized from different sources with variable chemical structures that allow different chemical functionalities [346]. NPs exhibit antimicrobial activities through targeting critical active sites in pathogens, leading to partial or complete inhibition [346]. Organic or inorganic (metal and metal oxide) NPs can be synthesized from different sources. Inorganic NPs possess bactericidal activity against bacteria using multiple mechanisms and, therefore, they are denominated “nanobactericides”. The nanobactericides activity of inorganic NPs is attributed to (1) their tiny size [346], (2) formation of weak and nonspecific interactions with bacterial surfaces [347], (3) Van der Waals forces (distance-dependent interactions between atoms or molecules) [348], and (4) attachment through specific receptor–ligand bonds [349]. Therefore, the bacterial cells’ susceptibility to NPs depends on their structural components as well as their growth rate [350]. Gram-positive bacteria are more susceptible to NPs than Gram-negative bacteria. Gram-positive bacteria have a permeable and negatively charged cell wall, making them an easy target for NP penetration, while the non-porous cell walls of Gram-negative bacteria serve as penetration barriers against the NPs [351]. Moreover, bacteria with slow growth rates are more sensitive to NPs than those with rapid growth rates. This is due to different stress-response genes’ expressions in fast-growing bacteria [350].

#### 4.7.1. Mechanisms of Action of NPs

**Figure 5:**
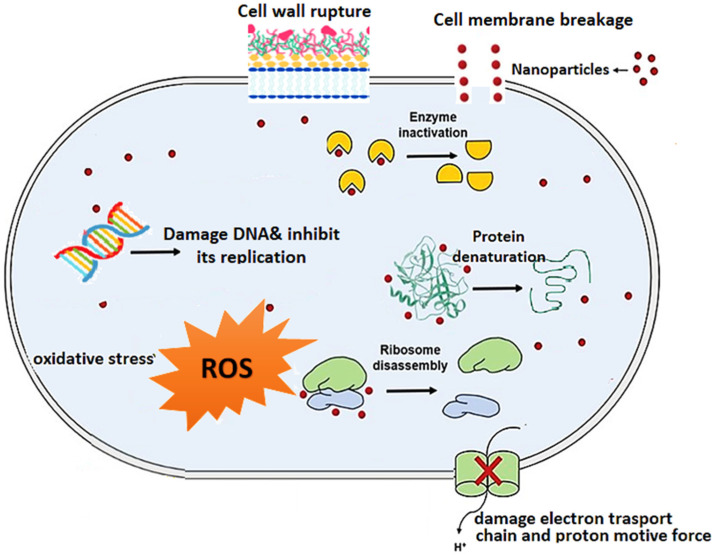
Mechanism of action of nanoparticles in bacterial cells.

The lethal impact of NPs on microbial cells is performed through (1) damaging the cell membrane and inhibition of permeability regulation due to direct attachment with the bacterial cell wall, (2) blocking electron transport and oxidative phosphorylation [352], (3) altering bacterial metabolism by interfering with enzymes, DNA and ribosomes, leading to protein and enzyme deactivation, prevention of DNA replication, and alteration of gene expression levels [353], (4) impeding the development of biofilms, (5) causing oxidative stress by releasing reactive oxygen species (ROS), and (6) excitation of host immune responses (Figure 5) [354,355]. Antimicrobial activities of NPs against different pathogenic bacteria, and mechanisms of action, are shown in Table 5.

#### 4.7.2. Limitations of NPs

The major limitations of NPs are (1) local and systemic toxic complications in the human body and potential inhibition activity on gut microbiota [350]; (2) silver NPs’ Ag accumulates in human organs such as colon, lung, bone marrow, liver, spleen, and the lymphatic system, leading to damage and/or decrease of organ efficacy and dysfunction [344] (additionally, Al_2_O_3_ NPs were reported to exhibit toxic effects on neurons [356]); (3) oxidative damage induced by CuO NPs, ZnO NPs, or TiO_2_ NPs [344,357]; (4) the buildup of metallic NPs in various tissues might cause renal damage, and liver or lung toxicity [358]; (5) the lack of a well-described standard technique that is not influenced by the properties of the NPs; and (6) that although resistance of bacteria to NPs rarely happens, some literature mentions that bacteria may develop NP resistance following exposure to metal NPs, such as Ag, Au, or Cu [359,360,361], or metal-oxide NPs, such as Cu^2+^ and Cu-doped TiO_2_ and Al_2_O_3_ NPs [362].

**Table 5:** Effects of nanoparticles on different pathogenic bacteria and the mechanism of their antimicrobial activity.

NPs	Particle Size	Target Bacteria	Mechanism of Action	Reference
Silver (Ag)	1–100 nm	*S. epidermidis*, MRSA, vancomycin-resistant *Enterococcus* (VRE), extended-spectrum beta lactamase (ESBL)-producing organisms, MDR *E. coli*, *P. aeruginosa*, *K. pneumoniae*, carbapenem and polymyxin B-resistant *A. baumannii*, and carbapenem resistant *P. aeruginosa, E. coli*	Generate reactive oxygen species (ROS), stopping cytochrome chains, membrane damage, dissipation of proton gradients, destabilization of RNA and DNA	[343,351,352,363]
Gold (Au)	1–100 nm	MRSA	Damage membranes and respiratory chains, inhibit ATPase activity, decrease the binding between tRNA and ribosomes and formation of pores in the cell wall	[344,351,352]
Copper (Cu)	2–350 nm	MDR *E. coli*, *A. baumannii*	Dissipation of cell membranes, generation of ROS, lipid peroxidation, protein oxidation, and DNA degradation	[343,364]
Silica (Si)	20–400 nm	MRSA	Generation of ROS and lysis of cell walls	[351,352]
Aluminum (Al)	10–100 nm	*E. coli*	Generation of ROS and lysis of cell walls	[344]
Iron oxide NP	1–100 nm	MDR *E. coli*, MRSA, *K. pneumoniae,*	ROS-generated oxidative stress: superoxide radicals (O^−2^), hydroxyl radicals (OH^−^), hydrogen peroxide (H_2_O_2_)	[351]
Titanium dioxide (TiO_2_)	30–45 nm	*E. coli*, *P. aeruginosa*, *S. aureus*, *E. Faecium*	ROS generation and adsorption to the cell surface	[344]
Zinc oxide (ZnO)	10–100 nm	*Enterobacter aerogenes*, *E. coli*, *K. oxytoca*, *K. pneumoniae*, MRSA, *K. Pneumoniae*, ESBL-producing *E. coli*	Generation of ROS, disruption of membranes, adsorption to cell surface, and damage to lipids and proteins	[365]
Magnesium oxide (MgO)	15–100 nm	*S. aureus*, *E. coli*	ROS generation, lipid peroxidation	[343]

### 4.8. Organic Acids (OAs)

Organic acids are widely used as antimicrobials in food processing and many industries [366]. Bacteria, fungi, and yeast play a critical role in the synthesis of organic acids during their lifecycle with high yields that can be achieved using cost-effective substrates. The bioproduction of OAs depends on many factors, such as the species of microorganism, inoculum size, substrate or carbon source, and environmental conditions (aeration, temperature, pH and agitation) [367]. Increasing the acidity by adding an acidulant or integrating natural fermentation is one of the commonly used methods to minimize and/or inhibit microbial growth. Using organic acids as an alternative to antibiotics depends on several factors, such as chemical formula structure, molecular weight, the value of the dissociation constant (pKa), minimum inhibitory concentration (MIC), nature of the microorganism, and exposure time to the food [368], where the pKa is an important criterion because of the undissociated part of the acid that is responsible for the antimicrobial effect.

Common OAs that are microbially produced and commercially used for microbial inhibition and food processing (Table 6) include (1) acetic acid, which is produced after fermentation of many substrates, such as glucose, lactose, and sucrose. This has the European code E260 and is used in the production of vinegar, stabilizer, flavor enhancer, and firming agent [367]. (2) Adipic acid is a crucial intermediate in the pathways of cyclic alkanes, long-chain aliphatic dicarboxylic acids and cyclic alcohols [369]. It is commonly used in the synthesis of polymers, plasticizers, nylon, clothing, automobile parts, and lubricant [370]. (3) Butyric acid is used in the fuel, plastic pharmaceutical, and textile industries. (4) Citric acid is used as a pH regulator, flavor enhancer, pharmaceutical reagent, and firming agent, in addition to its antimicrobial properties [371]. It is also used in soft candies, baked goods, gelatins, snacks, dairy products, and cheese warps as an antimicrobial and in the fuel industry. (5) Lactic acid is used in dairy products, biochemical processes, and the leather, pharmaceutical, textile, and biodegradable biopolymer industries [372]. (6) Malic acid, which is an intermediate compound in the tricarboxylic acid cycle, is naturally found in fruits including apricot, blackberry, cherry, mango, peach, and plum. It has been used in the food, water treatment, textile, metals, and pharmaceutical industries [373]. (7) Phenyl lactic acid naturally exists in honey and has an effective and broad microbial activity against bacteria, fungi, and yeast [374]. (8) Propionic acid naturally presents in apples, strawberries, grains, and cheese [375]. Adding propionic acid to chick diets was found to improve their growth, exert an antimicrobial effect in the intestine, and reduce the yellowness of the meat [376]. (9) Succinic acid is used in food preservation, perfume intermediates, herbicide production, and the plastics and textiles industries [377].

#### 4.8.1. Mechanisms of Action of OAs

The inhibitory mechanism of OAs is mainly due to the passage of the compound in proton-like form into the pathogen’s plasma membrane. When organic acid molecules pass through the cell membrane, they subsequently dissociate, resulting in the release of charged anions and protons that could not pass through the plasma membrane on their own [378]. It has been reported that these accumulating anions are poisonous and capable of blocking metabolic processes [379]. OAs limit microbial growth through altering lipophilic properties and, thus, allowing the uncharged form of weak acids to diffuse into the cytoplasm through the microbial plasma membrane until reaching an equilibrium. The decrease in the intracellular pH leads to microbial growth inhibition through denaturation of enzymes and structural proteins and DNA damage [380]. OAs were also reported to cause perturbation of membrane function by intercalation or chelation of ions essential to bacterial membrane stabilization [381]. There is also evidence that weak acids result in accumulation of anions inside the cytoplasm, which may have an osmotic effect and alter metabolic processes within the cells [380]. Furthermore, the inhibitory activity of mild organic acid might be due to an induced response involving an integral membrane protein (Hsp30) that strives to restore equilibrium. This protein inhibits the rise in membrane ATPase by serving as a ‘molecular switch’ to save the cellular energy reserves that the enzyme would otherwise utilize to establish equilibrium. However, this reaction is energy-intensive and the process reduces the available energy pools for growth and other vital metabolic functions [382].

#### 4.8.2. Limitations of OAs

The increase in OAs concentrations could change the sensory quality (color, odor, flavor, and taste) of preserved food [383]. Some pathogens possess several mechanisms to counteract the inhibitory impact of OAs, which may result in resistance to their antimicrobial activity and/or to the harsh acidic conditions [384]. Additionally, the procedure to get production approval from regulatory agencies is very complex and time-consuming [382]. Direct applications in living organisms, such as poultry, may reduce their growth performance because OAs are rapidly metabolized in the foregut [385].

**Table 6:** The structures of commonly used organic acids, their main producers, and their activity against various pathogenic bacteria.

Organic Acid (pKa1)	Chemical Structure	Main Microbial Producers	Active against	References
Acetic acid (4.76)	C_2_H_4_O_2_	*C. formicoaceticum*, *Acetobacter*, *Gluconobacter,*	*L. monocytogenes*, *S.* Typhimurium and *E. coli*	[367]
Adipic acid (4.41)	C_6_H_10_O_4_	*E. coli*	*Alternaria solani*, *Botrytis cinerea*, *Phytophthora capsici*, and *P. citrophthora*	[386]
Butyric acid (4.82)	C_4_H_8_O_2_	*C. butyricum*, *Butyrivibrio* sp., *Eubacterium* sp., *Fusobacterium*, *Megasphera* sp., *Sarcina* sp.	*S.* Enteritidis, *C. perfringens*, *E. faecalis*, and *S. pneumoniae*	[387,388]
Caprylic acid (4.89)	C_8_H_16_O_2_	*Mixculture* from brewery wastewater	*Vibrio parahaemolyticus & Dermatophilus congolensis*	[389]
Citric acid (3.13)	C_6_H_8_O_7_	*Aspergillus ficum,* *Acremonium*, *Bacillus*, *Bostrytis*, *Candida*, *Aschochyta*, *Eupenicillium*, *Debaromyces*, *Hansenula*, *Trichoderma*, *Mucor*, *Pichia, Saccharomyces*, *Talaromyces*, *Penicillium*, *Torulopsis*, *Yarrowia*, and *Zygosaccharomyces*	*Yersinia enterocolitica* *Shigella dysenteriae* *E. coli* O157:H7	[390,391]
Fumaric acid (3.02)	C_4_H_4_O_4_	*Rhizopus arrhizus*	*Talaromyces flavus*	[392]
Lactic acid (3.86)	C_3_H_6_O_3_	*Rhizopus oryzae,* *Aspergillus*, *Bacillus*, *Carnobacterium*, *Enterococcus*, *Escherichia, Lactobacillus*, *Lactococcus, Rhizopus*, *Saccharomyces*	*B. coagulans,* *L. monocytogenes*	[393]
Malic acid (3.40)	C_4_H_6_O_5_	*Ustilago trichophora*, *E. coli*, *Saccharomyces*, *Aspergillus* sp. and *Zygosaccharomyces Aureobasidium pullulans*	*L. monocytogenes*, *E. coli* O157:H7, *S.* Enteritidis and *S. gaminara,*	[394]
Phenyllactic acid (4.31)	C_9_H_10_O_3_	*B. coagulans,* *Lactobacillus*, *Enterococcus*, *Leuconostoc*, and *Weissella*, *Leuconostoc*, *L. plantarum* 1081, *L. acidophilus* 1063, *L. paracasei* 1501	*L. monocytogenes* *Aspergillus* spp. *Penicillium* spp.	[10,142]
Propionic acid (4.87)	C_3_H_6_O_2_	*Propionibacterium acidipropionici*	*L. plantarum*, *Sarcina lutea*, *S. ellipsoideus*, *Proteus vulgaris*, *S. aureus*, and *Torula* spp. *E. coli* K12 and *Salmonella*	[154]
Succinic acid (4.21)	C_4_H_6_O_4_	*Yarrowia lipolytica*, *Anaerobiospirillum succiniciproducens*, *Mannheimia succiniciproducens,* and *Actinobacillus succinogenes*	*S.* Typhimurium, *E. coli, B. subtilis*, and *S. suis*	[395,396]
Tartaric acid (2.98)	C_4_H_6_O_6_	*Gluconobacter suboxydans*	*L. monocytogenes*, *E. coli* O157:H7 and *S. gaminara*	[397]
Valeric acid (4.82)	C_5_H_10_O_2_	*Megasphaera elsdenii*	*C. jejuni*	[398]

### 4.9. Essential Oils (EOs)

Essential oils are volatile, aromatic, and oily liquids extracted from plant parts, such as seeds, leaves, buds, twigs, flowers, bark, herbs, wood, fruits, and roots [399]. Plants generate EOs as a natural defense against pathogens and herbivore feeding by reducing the appetite of herbivores. As a result, the Department of Health and Human Services has designated EOs as safe antibacterial additives [400]. To date, about 3000 EOs have been recorded, 300 of which are economically valued in the pharmaceutical, agronomic, food, sanitary, cosmetics and perfume industries [401]. EOs are complex natural mixes that contain anywhere from 20 to 60 distinct components in various proportions. The antibacterial effects of EOs are dictated by their primary ingredients (85%), which include terpenes, terpenoids, and aromatic and aliphatic groups from different natural sources [402]. These groups are characterized by low molecular weights, which are limonene (31%) and α-phellandrene (36%) in *Anethum graveolens* leaf oil, d-limonene (over 80%) in citrus peel oils, α/β-thujone (57%) and camphor (24%) in *Artemisia herba-alba* oil, carvacrol (30%) and thymol (27%) in *Origanum compactum* oil, α-phellandrene (36%) and limonene (31%) in *Anethum graveolens* leaf oil, menthol (59%) and menthone (19%) in *Mentha piperita* oil, and carvone (58%) and d-limonene (37%) in *Anethum graveolens* seed oil [403].

Menthol, pulegone, linalool, thymol and camphor, extracted from *Salvia lavandulifolia Lavandulaangustifolia*, *Mentha piperita*, *Mentha pulegium*, and *Satureja montana*, respectively, have shown antagonistic effects against *P. aeruginosa*, *S. pyogenes*, *S. mutans*, *S. sanguis*, *S. salivarius*, and *E. feacalis* [404]. Thymol and carvacrol, extracted from many sources, such as *Origanum compactum*, *Lavandula latifolia*, *Lavandula angustifolia*, *Rosmarinus officinalis*, *Origanum vulgare*, *Thymus vulgaris*, and *Thymus zygis* chemotype thymol, have shown activity against *S. aureus*, *C. hystoliticum*, *C. perfringens*, *E. coli* O157:H7, *S.* Typhimurium, *S.* Enteritidis, and *L. monocytogenes* [404,405]. Linalool, linalyl acetate α-terpineol, β-caryophyllene and nerol, produced by *Mentha citrata* Ehrh, have shown inhibitory effects against *P. aeruginosa*, *K. pneumoniae*, *E. coli* (DH5α), *E. coli* (MTCC 723) and *S.* Typhimurium, *S. aureus*, *S. epidermidis* and *S. mutans* [406]. Additionally, E-anethole, linalool, 1,8-cineole, α-pinene, camphor, camphene, menthol, menthone, and limonene, produced by *Ocimum basilicum*, *Rosmarinus officinalis*, *O. majorana*, *Mentha piperita*, *Thymus vulgaris*, and *Pimpinella anisum*, have shown activity against *C. perfringens* [407]. *Epilobium parviflorum*, *Salvia desoleana*, *S. sclarea*, and *Allium sativum* were reported to produce palmitic acid, linoleic acid and α-linolenic acid, which have shown an ability to inhibit *E. faecalis*, *S. aureus*, *P. aeruginosa*, *S. epidermidis,* and *E. coli* [408]. Moreover, cinnamomum was reported to produce cinnamaldehyde, which was shown to inhibit *E. coli*, *S. aureus,* and *S.* Typhimurium [409]. Dipterocarpus gracilis was reported to produce elemicin and geranyl acetate, which were shown to suppress *B. cereus* and Proteus mirabilis [410].

#### 4.9.1. Mechanisms of Action of EOs

EOs and their components are characterized by their hydrophobic nature that allows them to interact with the lipids of the microbial cell membrane [411]. They can sensitize cells and cause severe membrane damage, resulting in leaking of essential intracellular contents, bacterial cytoplasmic membrane collapse, and bacterial cell death. Cell wall breakdown, cytoplasmic membrane damage, cytoplasm coagulation, and membrane protein degradation are the common causes of the leakage [412]. EOs also directly target biofilm formation. A recent study has shown that EOs can limit biofilm formation by binding to them, in addition to reducing cell-wall-related virulence factors and the translation of particular target microorganism regulatory gene products [413]. EOs can also operate as transmembrane carriers by swapping their hydroxyl protons for potassium ion, causing the electrical potential and the pH gradient across the membrane to dissipate. They also result in a reduction in proton motive force and depletion of intracellular adenosine triphosphate (ATP) pools. Potassium deficiency can also be troublesome, since it is necessary for the activation of several cytoplasmic enzymes, the maintenance of osmotic pressure, and intracellular pH regulation [413]. A phenolic group in EOs exhibits a direct antibacterial activity by altering bacterial membrane permeability and energy production. Moreover, the hydroxyl groups of EOs are thought to attach to bacterial proteins and block the function of amino acid decarboxylases in *E. aerogenes* [405].

#### 4.9.2. Limitations of EO Applications

Application of EOs in food processing reduces the availability of EOs as antimicrobial agents because food constituents contain many fatty, proteinaceous, and sugary components that may interfere with the action of EOs. Additionally, the concentration and the dose of EOs added to the food is 10–100 times lower than the in vitro concentrations, which may consequently result in a decreased efficiency of these EOs [414]. Moreover, addition of EOs in the food industry even at a low concentration may change the physical properties of the food product, such as odor and taste [415]. Since the optimum antimicrobial activity of EOs is at an acidic pH, many EOs are sensitive to high pH values [416]. The relationships between the toxicity of bioactive components of Eos, their chemical structures and functional groups, the influence of hydrophobicity, and the makeup of the microbial lipid membrane should be investigated extensively before EOs are used [415].

### 4.10. Fecal Microbial Transplant (FMT)

FMT is a process of transferring processed fecal material from the intestine of a healthy donor to the intestine of a recipient patient [417]. Processed fecal matter can be administered to the recipient through several methods, such as a nasoduodenal tube [418], nasojejunal tube [419], colonoscopy, or retention enema [420]. Colonoscopy administration of fresh or frozen and thawed fecal matter from stool banks into the cecum and colon of *C. difficile*-infected children in Maryland resulted in complete resolution of CDI in recipients as well as reductions in both AMR and multidrug resistance genes. Moreover, FMT resulted in sustained elevations in alpha diversity post-FMT as well as significant changes in beta diversity, in addition to improving the biosynthetic pathways [420]. In another study performed in mice, when a combination of FMT and lytic phages was used for treatment of *S.* Typhimurium, a complete clearance of *Salmonella*, a reduction in inflammatory cytokines, and restoration of the intestinal microbial diversity was observed [421]. Additionally, patients with MRSA enteritis who were treated with FMT through nasointestinal tube, jejunostomy fistula tube or gastrostomy fistula tube had negative stool cultures for MRSA, and gut microbiota analysis also revealed that all recipients developed donor-related bacterial diversity [422]. FMT has also shown satisfactory results when given to patients infected with beta-lactamase-producing Enterobacteriaceae. Four weeks after the first FMT dose through nasoduodenal tube, decolonization was detected in 20% of the recipients, with recipients’ microbial composition showing a shift toward the donors’ microbial diversity [423]. Administration of fresh fecal matter using nasojejuneal tube into *K. pneumoniae*-infected patients stopped sepsis and resulted in elimination of *K. pneumoniae*, as demonstrated in the blood cultures and general improvement of the health status. Moreover, a restoration of microbial diversity was observed after 6 weeks of treatment [419]. Administration of fresh fecal matter using nasoduodenal gastroscope into the third part of the duodenum of chronically infected hepatitis B patients (CHB) also resulted in suppression of the hepatitis B virus as well as clearance of the HBeAg [418]. In addition to its use in the treatment of microbial infections, FMT has also shown potential to improve non-infectious GIT conditions, such as ulcerative colitis, where it resulted in a lowered pediatric ulcerative colitis activity index (PUCAI) following completion of the FMT treatment course [424]. Similar observations were made for other inflammatory bowel diseases [425]. Moreover, several experiments were undertaken to assess the safety and efficacy of FMT in treating other extra-intestinal disorders, such as obesity and metabolic disorders [426] and psoriatic arthritis [427].

#### 4.10.1. Mechanisms of Action of FMT

Although the mechanism of treatment by FMT is not fully clear yet, it has been noticed that the restoration of healthy gut microbial diversity [428], including *Firmicutes* in recipients after FMT [428], is associated with improvement in the recipients’ health status [429]. It has been hypothesized that restoration of gut beneficial microbes, such as *Roseburia hominis* and *Bacteroides ovatus*, inhibits the pathogens’ growth [428] or competes with the pathogens for nutrients and growth environment [428]. The restored commensal microbes compete with *C. difficile* directly through competitive niche exclusion or indirectly through production of bacteriocins, such as thuricin CD [430].

#### 4.10.2. Limitations of FMT

While the development of FMT may seem easy [431], there are still some challenges associated with its preparation. These challenges include donor selection, stool processing, method of sample administration, colonization resistance, and relapse of infection. The donor for FMT has to be healthy, free from autoimmune, metabolic, and malignant disorders as well as pathogenic microorganisms [417]. It was noticed that microbiota from donors encounter resistance to colonizing the recipient’s intestine from the recipient’s gut flora, thus preventing them from performing their function [432]. Therefore, it is recommended that the donor should be one of the recipient’s relatives to achieve what is called “donor–recipient microbial matching” to overcome colonization resistance [433]. Additionally, naso-gastric administration is associated with respiratory side effects, while diarrhea is a common adverse effect of a colonoscopy [434]. Processing of a donor’s stool under aerobic conditions diminishes its quality, as it affects bacterial diversity by inhibiting the viability of normal anaerobes in the sample while inhibiting bacterial ability to produce short-chain fatty acids, which are crucial processes for homeostasis [432,435]. However, it is noteworthy that processing and freezing storage duration did not significantly impact the efficacy of FMT in *C. difficile* infection (CDI) [436]. For better FMT outcomes, proper site administration should be applied at the site of dysbiosis [432]. Relapse of infection is also one of adverse effects of FMT, as seen in about 20% of FMT-treated CDI [428]. It can be concluded that, to date, there is no perfect approach without adverse effects for FMT application. More studies concerning safety and efficacy are required to approve FMT for wide use in treatment.

### 4.11. Vaccines

Vaccines are preparations used to stimulate the body’s immune response against diseases by exploiting the ability of the human immune system to respond to, and remember, the antigens of pathogens. Several vaccines have been developed to make a revolutionary change in the world, such as fowl (avian) cholera, anthrax, polio, norovirus, rift valley fever, and rabies vaccines [437,438,439]. Vaccines play a pivotal role in reducing the need for antibiotics and controlling the emergence of AMR bacterial strains [440]. Vaccines reduce the burden of antimicrobial resistance through disease prevention and thus reducing the use of antibiotics [441,442]. This occurs as a vaccine curbing the ability of the pathogen to establish a foothold in the host, by conferring immunity against these pathogens, thus minimizing the chances of some bacterial mutations and the development and spread of resistant genes to other bacteria [443,444]. For instance, a 67% reduction in the circulation of penicillin-resistant invasive pneumococcal strains was demonstrated in a group of children that received pneumococcal conjugate vaccine 9 (PCV9) compared to controls in South Africa [445]. Conjugate vaccines combine weak antigens with strong antigens (which serve as the carriers) to increase the response of the body to the weaker antigen. In this context, the typhoid conjugate vaccine (TCV) has been introduced in children in order to protect them extensively from drug-resistant *S.* Typhi [446]. It has been observed that TCV can avert 44% of typhoid cases, of which 35% are resistant to antibiotics [447]. Salmonellosis, caused by *Salmonella* spp., is one of the most common zoonotic diseases associated with consumption of dairy and beef [448]. *S. enterica* serotype Dublin, which infects cattle and can be shed in milk, colostrum, and feces, also poses a threat to public health. *S.* Dublin causes bloodstream infections in humans, with a relatively high case fatality [449]. Data from the CDC (Centers for Disease Control and Prevention) showed that *Salmonella* Dublin infections caused more hospitalization during 1996–2004. Additionally, a higher percentage of *Salmonella* isolates were resistant to more than seven classes of antimicrobial drugs during 2005–2013 (50.8%) compared to only 2.4% during the period 1996–2004. Resistant *S. enterica* causes at least 100,000 foodborne human infections annually [450]. A commercial modified-live *Salmonella* Dublin vaccine (EnterVene-d) is approved by the USDA for use in calves, but vaccination does not reduce the likelihood of contamination or the risk to public health; it only improves clinical outcome [451]. *C. perfringens* enterotoxin (CPE) and Shiga-toxin-producing *E. coli* (STEC) are also common causes of food poisoning. Research has been conducted on possible development of a vaccine for CPE and STEC in the form of a bivalent food poisoning vaccine. The bivalent vaccine uses a fused protein (Stx2B-C-CPE) consisting of the B subunit of *E. coli* Shiga toxin 2 fused to CPE to enhance its antigenicity [452]. Two other extremely important bacteria, *C. jejuni* and *C. perfringens,* have been the subject of many vaccine studies in poultry [453,454,455,456]. The efficacy and commercial potential of these vaccines has been described and reviewed in detail elsewhere [457]. It is worth mentioning that the conserved N-glycan heptasaccharide conjugated to GlycoTag, or fused to the *E. coli* lipopolysaccharide core, has shown tremendous potential to reduce *C. jejuni* colonization in the gastrointestinal tract of chickens by up to 10 log_10_ [458]. Despite the demonstrated efficacy of this vaccine, its commercialization remains murky.

Vaccination can cause indirect effects on infections. While resistance is a predictable outcome of antibiotic use, resistance to vaccines is very rare [459]. Vaccines are administered prophylactically, whereas antibiotics are administered only once symptoms have begun to show. Thus, by the time antibiotics are administered, there are possibly already millions of copies of the pathogen, raising the probability of mutation occurring. Vaccines prevent the pathogen from gaining a foothold and multiplying in the first place. In many cases, the use of vaccines has globally eradicated some diseases, while decreases of 95% in the incidence of diseases like diphtheria, tetanus, and pertussis have been observed [460]. Much progress has been made in bacterial vaccine development. Bacterial vaccines can and should help address the global AMR problem. It is reasonable to believe that reductions in MDR infections as well as the prevention thereof can be achieved using bacterial vaccines. Attention should also be paid to the role of veterinary bacterial vaccines to reduce antibiotic use in animals, especially food-producing animals. The role of bacterial vaccines is set to expand dramatically in response to the crisis of AMR and MDR [461]. Although vaccines against major AMR pathogens are still missing, predictions of the impact of vaccines against AMR hint that vaccines could have a significant impact in controlling resistance [462].

#### 4.11.1. Mechanisms of Action of Vaccines

Vaccine types are quite varied in their formulations and mechanisms of action. The mechanisms through which different types of vaccines work include: (1) live, attenuated vaccines: these vaccines contain a live version of the pathogen that has been attenuated or weakened to the point where it loses its pathogenicity, but is still capable of inducing an immune response [463]; (2) killed whole-cell vaccines: the pathogen is killed or inactivated by treatment with gamma irradiation or a chemical agent; this preserves the structure of the epitopes but removes the pathogen’s ability to replicate or be virulent [464]; (3) toxoid vaccines: the pathogen’s toxin is purified and treated with formalin to destroy its toxic activity, while retaining enough antigenic activity to protect against disease [465]; (4) subunit vaccines: these contain protein or glycoprotein components of a pathogen that are able to induce a protective immune response [466]; outer membrane vesicles (OMVs) are comprised of bacterial outer membrane constituents naturally released from Gram-negative bacteria, and contain key antigenic components that can elicit a protective immune response but cannot cause disease [467]; (5) protein–polysaccharide conjugates: conjugate vaccines are composed of covalently linked bacterial polysaccharides to proteins; polysaccharides on their own do not elicit T-Cell response, whereas polysaccharides linked to certain proteins do elicit a T-cell response [468]; (6) recombinant viral and bacterial vector vaccines: these use harmless bacteria or viruses as vectors to introduce the genetic code of the antigens of the pathogen to the cells, to train the immune response [469]; and (7) nanovaccines: a new generation of vaccines using NPs as carriers and/or adjuvants; nanovaccines could target the area of the body where the disease originated from, while other vaccines target the whole body [470]. Table 7 shows examples of vaccines that have been developed and approved or are still currently being developed against foodborne bacterial pathogens in humans and livestock.

#### 4.11.2. Limitations of Vaccines

(1) Highly variable pathogens pose a challenge, as their genetic diversity within and between hosts make it difficult to identify an antigen that can be used for vaccine development [477]. (2) Vaccine failure is always a risk. This refers to an organism contracting an illness despite being vaccinated against it. This is usually due to individual immune response differences [478]. (3) Live attenuated vaccines usually need the cold chain to stay potent, which adds extra cost that especially affect developing countries that lack widespread refrigeration [463]. (4) Killed whole-cell vaccines lead to a weaker immune response than live vaccines, thus requiring many booster doses to maintain immunity [463]. (5) Toxoid and subunit vaccines usually require adjuvants and several doses because they are not highly immunogenic. High doses may lead to toxoid tolerance [479]. (6) Virus-like particle (VLP) vaccines are multimeric structures with no viral genome, making them very unstable. The levels of expression of VLP vaccines in different platforms vary greatly [480]. (7) OMV vaccines have an incredibly low yield because they are released spontaneously by bacteria in low quantities. The key antigens on their surface can also be in low quantities [481]. (8) Polysaccharide–protein conjugate vaccines have inconsistent numbers of conjugates in each batch, affecting vaccine efficacy. The carrier protein k, and the linker between the polysaccharide and carrier protein, may also be immunogenic and trigger an immune response against itself [482]. (9) Bacteria, such as *Streptococcus pyogenes* and *S. aureus*, develop AMR gradually and with little selective pressure [442]. Similarly, *Bordetella pertussis* shows AMR towards unprotected or partially protected people, due to incomplete vaccine protection [461]. Acellular vaccines against *B. pertussis* provide shorter protection than whole-cell vaccines [445]. Collectively, vaccination has a role in specific cases, and should be used in combination with other approaches to manage infection or lower the demand for antibiotics [462].

### 4.12. Antibodies

Antibodies, also known as immunoglobulins, are the most diverse set of proteins [483]. They have two major functions: antigen binding and effector functions [484]. Most of these effector functions are induced via the constant Fc (fragment crystallizable region, the tail region of an antibody) of the antibody, which can interact with complementary proteins and specialized Fc-receptors. This can activate or inhibit pathways, depending on the type of receptor [485]. Therapeutic antibacterial monoclonal antibodies (mAbs) are gaining traction as an alternative in treating infectious diseases [442]. Monoclonal antibodies could offer more effective ways of addressing antibiotic resistance and bacterial infections due to their superb specificity, by which they target conserved pathways. This allows for fewer off-target effects and less selective pressure for cross-resistance to other mAbs or antibiotics. Monoclonal antibodies also do not harm the beneficial microbiome [486,487] In 1879, Amil von Behring and Shibasaburo Kitasato were the first to develop antibodies called antitoxins that target specific toxins. Blood-serum-containing antitoxin was directly injected to convey immunity to diphtheria in humans [488,489]. The toxin/antitoxin approach provided a steady treatment against numerous pathogens, such as *Haemophilus influenzae*, *Neisseria meningitides*, *Corynebacterium diphtheria*, *Clostridium tetani*, *S. pneumonia,* and Group A *Streptococcus*. However, the antitoxin approach exhibits heterogeneity between lots, allergic reactions, and a limited spectrum, eventually leading to its replacement by antibiotics in 1930 [490]. Antibiotic production peaked for the next 80 years because of their safe application and their ease of formulation and manufacture [491]. However, due to the development of the hybridoma technology and recent advances in mAb engineering, awareness has shifted back to antibacterial mAbs [492].

#### 4.12.1. Mechanisms of Action of mAbs

**Figure 6:**
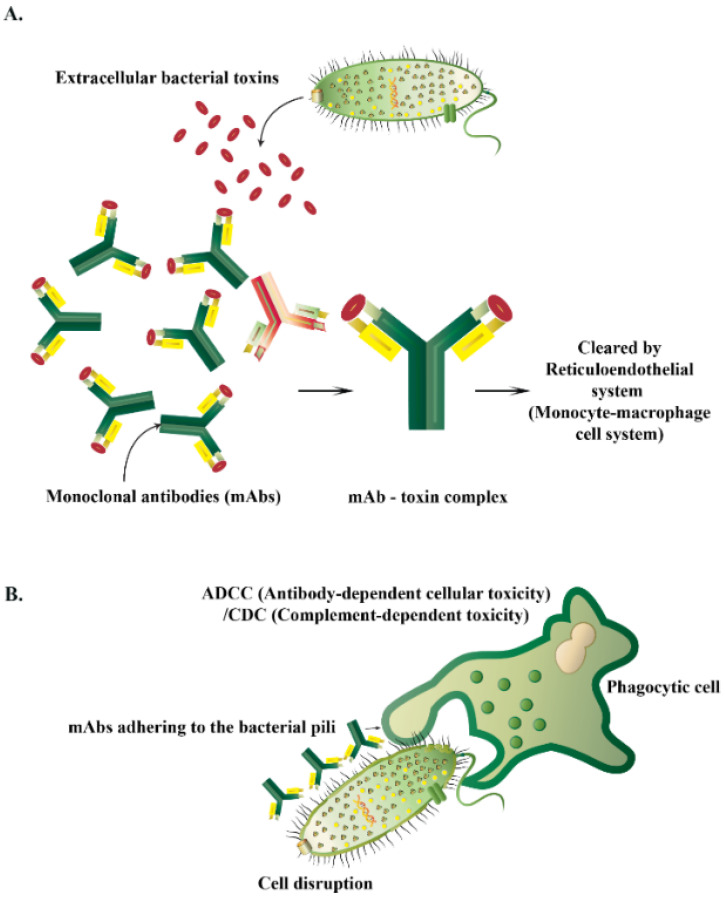
Monoclonal antibodies’ (mAbs) mode of action. (**A**) Depicts the neutralization of bacterial toxin by mAbs. Bacterial toxins are secreted by bacteria. The antibodies attach to the toxins and get cleared by the reticuloendothelial system. The reticuloendothelial system is a cluster of phagocytes that clear matter. (**B**) mAbs adhere to the pili structures of the bacteria. Pili are hair-like appendages found on the surface of many bacteria that function to attach cells to surfaces. The antibodies attach along with the phagocytic cells (antibody-dependent cellular cytotoxicity—ADCC) and/or complement (complement-dependent cytotoxicity—CDC) and eventually cause the bactericidal effect.

mAbs provide their anti-virulence effect through the following mechanisms: (1) mAbs binding to their target antigen: This can be a soluble ligand or a receptor; either way, the interaction is blocked between the ligand and receptor. This can also lead to internalization of receptors or apoptosis of the targeted cells [486]. (2) Blockage of the bacterial virulence factors (bacterial toxin neutralization): Neutralization of the toxin occurs when it binds to the mAb and forms an mAb–toxin complex. This complex eventually gets cleared by the reticuloendothelial system (Figure 6A). Monoclonal antibodies may also bind to the structural components of the cell surface, invoking immune-system-dependent cytotoxicity or direct bactericidal effects [493]. This also limits collateral damage, such as development of drug resistance, as mAbs often target virulence proteins rather than proteins required for survival, leading to lower virulence [494], while aiding both the host’s adaptive and innate immune systems.

Several bacteria, such as *Bordetella pertussis*, *V. cholerae*, *Bacillus anthracis*, *C. diphtheriae*, *C. botulinum*, *C. tetani*, *C. difficile*, *C. perfringens*, *Salmonella* spp., and EHEC, secrete disease-causing toxins to which mAbs can bind [488]. Other virulence factors such as type III secretion systems (T3SS), adhesins, and pili, along with outer membrane transporters, which are exposed on the bacterial cell membrane, have also been identified as potential antibody targets. mAbs targeting these antigens cause bactericidal effects (Figure 6A) [495]. Ideally, these toxins (antigens) should be in abundance to allow the mAbs to bind to them specifically, avoiding any off-target binding. Additionally, the binding of these mAbs does not cause immediate bactericidal effects. Their action is closely associated with the phagocytic cells (antibody-dependent cellular cytotoxicity—ADCC) and/or complement (complement-dependent cytotoxicity—CDC), which eventually causes the bactericidal effect [496] (Figure 6B).

Many different mAbs are currently in clinical trial phases or have already been approved. For example, the isotype IgG1(κ) human monoclonal antibody auvratoxumab (MEDI-4893) targets pneumonia-causing *S. aureus* alpha-hemolysin toxin and neutralizes it, thereby preventing its colonization. This approach is much more effective than antibiotics because the use of antibodies is not expected to lead to resistance in the future. In addition to this, antibodies work irrespective of the antibiotic resistance status of the pathogen [494]. A new antibody–antibiotic conjugate (DSTA4637S) was developed to target intracellular *S. aureus.* It consists of anti-*S. aureus* thiomab human immunoglobulin G1 (IgG1) monoclonal antibody linked to a novel rifamycin-class antibiotic [497]. Following its phagocytosis by phagocytic cells, the intracellular cathepsins cleave the link between the mAbs and antibiotic, resulting in release of a rifamycin-class antibiotic, which subsequently kills the intracellular *S. aureus* [497].

Co-mixtures of IgG1 human monoclonal antibodies with efficacy against botulinum toxin, produced by *C. botulinum*, (BoNT) serotypes A (BoNT/A, NTM-1631) and B (BoNT/B, NTM-1632), were developed [498]. Their mode of action involves high-affinity binding of the mixture to the toxin, blocking cellular binding epitopes on the toxin, and increasing hepatic clearance of the toxin–Ab immune complexes. The results revealed that NTM-1632 does not bind human epitopes, which means that it is less likely to have off-target effects and can be used post-exposure without having any negative consequences [498]. Similarly, Obiltoxaximab (Anthim^®^, ETI-204) is a monoclonal IgG1(κ) antibody that is being developed for the prevention and treatment of *B. anthracis* and works by neutralizing the free protective antigen of *B. anthracis*, thereby inhibiting the toxin [499]. Another mAb, raxibacumab (ABthrax) is a human IgG1(λ) monoclonal antibody that is capable of neutralizing lethal antigens of *B. anthracis*, inhibiting cell death [500]. A human chimeric monoclonal antibody (isotype IgG), pagibaximab, is currently in phase II of clinical trials for treatment of *S. epidermidis* (lipoteichoic acid) causing staphylococcal sepsis. Pagibaximab enhances serum opsonophagocytic activity, making all staphylococci opsonizable [501]. In addition, setoxaximab/pritoxaximab are mouse/human chimeric IgG1(κ) antibodies that target Shiga toxin 1 and Shiga toxin 2, produced by *E. coli*. The combination is named Shigamab and developed for the treatment of hemolytic–uremic syndrome (HUS). The neutralizing mAbs target their specific Shiga toxin, eliminating it from circulation [502]. Shigamab was found to be safe and well tolerated in phase I and II clinical studies. An additional pre-clinical study has also been completed in a HUS baboon model, in which Shigamab was shown to protect the animals against a lethal dose of toxin when administered up to 48 h post-intoxication. A human IgG1 monoclonal antibody, Aerucin, is being developed by Aridis Pharmaceuticals. Aerucin targets *P. aeruginosa* alginate using opsonophagocytosis as the mechanism of action. Specific OprF/I IgG antibodies were detected in all IC43 administered groups. From day 0 to day 14, a four-fold or more increase in the antibody titers was observed in >90% of subjects. At 90 days, titers started to decline but remained higher than the placebo groups for up to six months [503]. Additionally, DSTA4637S was safe and well tolerated in healthy volunteers in the phase 1 single-ascending-dose study. DSTA4637S for *S. aureus* infections is safe and has a favorable PK profile [497]. However, antibody–antibiotic conjugates can still increase the incidence of AMR, whereas pure mAb treatments, such as suvratoxamab, are not likely to. In terms of *P. aeruginosa,* a phase II clinical trial (NCT03027609) of Aerucin saw no significant difference between Aerucin and placebo patient groups for treatment of *P. aeruginosa* patients, whereas panobacumab improved clinical outcome in a short time [504].

#### 4.12.2. Limitations of Abs

Despite the undeniable impact of mAbs in controlling many diseases, there are still some challenges concerning mAbs: (1) Production expenses: currently, mammalian cells, which allow human-like N-glycosylation and other post-translational modifications, are used to produce mAbs. This requires specialized eukaryotic machinery produce mAbs in the active form [505]. Mammalian cells also have several drawbacks when it comes to bioprocessing and scale-up, which results in long processing times and elevated costs. Moreover, high doses of the antibodies are required to reach clinical efficacy [506]. These factors limit the availability of the wide use of mAbs. (2) Systemic administration of mAbs is unsuitable for non-invasive routes of administration, such as oral, nasal, or pulmonary, as they are susceptible to chemical and enzymatic degradation in the gastrointestinal tract. In murine models, mAbs have been shown to largely remain in the blood.

Only about 20% of the administered dose reached the target tissue. Penetration and retention in the target area rely on the characteristics of the mAbs, such as molecular size, shape, affinity and valency [505]. By using methods such as in vivo gene transfer, costs can be greatly reduced, as one injection can produce mAbs in vivo long-term [507]. However, hybridoma mAb technology carries with it the risk of cancer, as the cell lines used are immortalized using the Epstein–Barr virus. There is also the risk of contamination of different cell lines, genetic instability of the cell line, and consistency and level of the expression and stoichiometric ratio of both the heavy and light chains [208,508]. Imbalances in the chain production can be toxic to the cell [509]. Nonetheless, mAbs remain one of the most promising technologies in the age of growing AMR threats.

### 4.13. Conclusions and Future Perspectives

The alarming rise in the emergence and spread of AMR and the associated global impact necessitate an urgent intervention of alternatives to combat the growing threat of antibiotic-resistant bacteria. Beside their potential adverse events, the inappropriate prescription or dispensing of antibiotics for humans and their irrational use in animal agriculture are among the factors contributing to the growing incidence of AMR in humans. As such, averting AMR could be achieved by focusing on two aspects: one is to implement antimicrobial stewardship programs through promoting prudent use of antibiotics in healthcare and agricultural settings, and the other is to develop effective antimicrobial alternatives to substitute antibiotics in animal food production. In fact, developed countries, including Europe and North America, have taken steps to ban the use of sub-therapeutic doses of antibiotics as growth promoters in livestock and poultry production; however, such steps have yet to be implemented in developing countries. Thus, a global solution is crucial to tackle AMR, as the world has become increasingly interconnected. Research efforts have been made to limit AMR in both humans and animals by exploring various interventions, including SMs, QSIs, probiotics, prebiotics, phage therapy, nanoparticles, EOs, AMPs, OAs, FMT, vaccines, and immune-based strategies, as potential replacements for antibiotics. Despite the promising role of most of these strategies in promoting host immunity and in antagonizing a range of human and animal pathogens, their variable effects, combined with their limited spectrum, safety concerns, and poor efficacy, are among the potential limitations to their use. Nonetheless, the exuberant development of molecular technologies may improve the efficacy of existing strategies and reduce their limitations. For instance, the recent breakthroughs in CRISPR-Cas9-based genome editing offer a revolutionary platform for designing safe and effective vaccines. Likewise, computational molecular biology has directed vaccine development towards genome-based reverse vaccinology approaches, a process of analyzing the whole genome sequence for identification of novel target antigens. The processes of 16S rRNA next-generation sequencing and bioinformatics analysis have enabled the identification of bacterial strains at species level. This technology can be utilized to not only identify novel probiotic species, but also to develop a consortium of beneficial microbes, which may offer a safer and acceptable alternative to FMT. With millions of people travelling around the world and the uncontrollable spread of AMR, holistic AMR control requires global solidarity to expand and implement robust antimicrobial stewardship programs in both medical and veterinary practices.

## Figures and Tables

**Table 7:** Vaccines against infectious bacterial pathogens, either approved or in the research stage.

Vaccine Name	Target Pathogen	Indicated for Use in	Notable Observations	Reference
M01ZH09 vaccine Live attenuated *S.* Typhi, strain *S.* Typhi ZH9	*S.* Typhi	Humans	Vaccine extremely well tolerated. Adverse events did not differ between cohorts or from subjects receiving placebo; M01ZH09 was highly immunogenic in all dose ranges. Serologic responses measured by *S.* Typhi LPS-specific IgA and IgG ELISA were seen in most volunteers at all dose levels and time points post-vaccination.	[471]
Whole cell *S. aureus* vaccine (StartVac)	*S. aureus*	Cattle	The 45% observed reduction in the basic reproduction ratio of *S. aureus* is encouraging, but highlights that vaccination is only an additional tool in the control of *S. aureus* infections on dairy farms. Efficacy was dependent upon the age group of the animals, where first-lactation animals showed a higher value. Compared with animals in third and higher lactation.	[472]
Whole cell *S. aureus* vaccine (Lysigin)	*S. aureus*	Cattle	Lower mean duration of clinical mastitis. No evidence that the vaccine reliably prevented *S. aureus*, but Lysigin showed a benefit in reducing the clinical severity and duration of clinical disease after challenging.	[473]
Modified live *S. dublin* vaccine (EnterVene-d)	*S. enterica* serotype Dublin	Cattle	The vaccine induced the immune response via stimulation of cell-mediated, humoral, and mucosal immunity. Calves that received colostrum from vaccinated cows had significantly higher *S.* Dublin titers compared to calves born to unvaccinated cows.	[474]
Alpha toxin (CPA) toxoid vaccine (NetVax)	C. *perfringens*	Poultry	Overall, the vaccine appeared to be safe, with no observed systemic reactions or adverse effects on performance or reproduction. Vaccination of broiler breeder hens induces the production of antibodies in the circulation of the hen, which remain at significant levels throughout the laying cycle. Antibodies are transferred from the hen to egg yolk, resulting in antibodies in the circulation of 7-day-old chicks.	[475]
N-glycan-based vaccine *	*C. jejuni*	Poultry	Reduce the cecal *Campylobacter* by 6 log_10_	[458]
AviPro Megan Vac 1	S. Typhimurium, *S.* Enteritidis and *S. Heidelberg*	Poultry	MV1 was effective at reducing cecal *S.* Enteritidis counts. The live attenuated vaccine had the added advantage of not persisting in the chicks.	[476]
PLGA-encapsulated CpG ODN and *C. jejuni* lysate	*C. jejuni*	Poultry	Reduced *C. jejuni* colonization by up to 2.4 log_10_, modulated intestinal immune responses, modulated the gut microbiome composition, enhanced the production of *C. jejuni*-specific antibodies	[453,454,456]

* Still in the research and development stage.

## Data Availability

Not applicable.
